# Histone Deacetylase Complex 1 and histone 1 epigenetically moderate stress responsiveness of *Arabidopsis thaliana* seedlings

**DOI:** 10.1111/nph.19165

**Published:** 2023-08-10

**Authors:** Giorgio Perrella, Carlo Fasano, Naomi A. Donald, Loretta Daddiego, Weiwei Fang, Damiano Martignago, Craig Carr, Lucio Conti, Pawel Herzyk, Anna Amtmann

**Affiliations:** ^1^ Department of Biosciences Università degli Studi di Milano Via Celoria 26 Milan 20133 Italy; ^2^ Plant Science Group School of Molecular Biosciences (SMB), University of Glasgow Glasgow G12 8QQ UK; ^3^ Italian National Agency for New Technologies, Energy and Sustainable Economic Development Trisaia Research Centre Rotondella (Matera) 75026 Italy; ^4^ Glasgow Polyomics, Wolfson Wohl Cancer Research Centre University of Glasgow Glasgow G61 1QH UK

**Keywords:** Arabidopsis, germination, histone modifications, salt stress, transcriptional regulation, transcriptomics

## Abstract

- Early responses of plants to environmental stress factors prevent damage but can delay growth and development in fluctuating conditions. Optimising these trade‐offs requires tunability of plant responsiveness to environmental signals.
- We have previously reported that Histone Deacetylase Complex 1 (HDC1), which interacts with multiple proteins in histone deacetylation complexes, regulates the stress responsiveness of Arabidopsis seedlings, but the underlying mechanism remained elusive.
- Here, we show that HDC1 attenuates transcriptome re‐programming in salt‐treated seedlings, and we identify two genes (*LEA* and *MAF5*) that inhibit seedling establishment under salt stress downstream of HDC1. HDC1 attenuates their transcriptional induction by salt via a dual mechanism involving H3K9/14 deacetylation and H3K27 trimethylation. The latter, but not the former, was also abolished in a triple knockout mutant of the linker histone H1, which partially mimics the hypersensitivity of the *hdc1‐1* mutant to salt stress. Although stress‐induced H3K27me3 accumulation required both H1 and HDC1, it was not fully recovered by complementing *hdc1‐1* with a truncated, H1‐binding competent HDC1 suggesting other players or independent inputs.
- The combined findings reveal a dual brake function of HDC1 via regulating both active and repressive epigenetic marks on stress‐inducible genes. This natural ‘anti‐panic’ device offers a molecular leaver to tune stress responsiveness in plants.

## Abstract 2

See also the Commentary on this article by Archacki, **241**: 7–9.

## Introduction

Plants regulate germination and development at various stages to ensure the best success under challenging conditions (Nicotra *et al*., 2010; Lee *et al*., 2012; Kaiserli *et al*., 2018). When young seedlings perceive abiotic stress such as drought or salinity, they halt the extension of radicles and the development of cotyledons (Jakab *et al*., 2005; Cutler *et al*., 2010; Daszkowska‐Golec, 2011). This ‘wait‐and‐see’ strategy ensures survival, but repeated interruption of development can hamper plant progression when conditions fluctuate. In a field scenario with reoccurring moderate stress events early in the season, yields could potentially be optimised by preventing unnecessary responses. It is, therefore, important to obtain a mechanistic understanding of the processes that underpin the stress sensitivity of young seedlings.

The re‐programming of development upon perception of environmental signals is mediated through elaborate signalling networks involving transcriptional regulation of a plethora of genes (Baroux *et al*., 2011; Song *et al*., 2012; Asensi‐Fabado *et al*., 2017). Transcriptional activation and repression of genes occur in the context of chromatin, the assembly of DNA with nucleosomes comprised of histones H2A/B, H3 and H4 and the linker histone H1 (Martienssen & Colot, 2001; Goldberg *et al*., 2007; Kouzarides, 2007; Kawashima & Berger, 2014). Histone modifications such as de‐/acetylation of lysine residues play a fundamental role in altering transcriptional responses through modification of chromatin structure and recruitment of regulatory proteins (Kim *et al*., 2015, 2017; Ueda & Seki, 2020). Similar to their counterparts in yeast and mammals, plant histone deacetylases (HDACs) operate as part of multi‐protein complexes (Pandey *et al*., 2002; Mehdi *et al*., 2016; Ning *et al*., 2019), including co‐repressors and histone‐binding proteins, but their exact composition remains to be fully elucidated and is likely to vary between developmental stages and environmental conditions.

We have previously identified Histone Deacetylase Complex 1 (HDC1) as a component of HDAC complexes in *Arabidopsis thaliana* (Perrella *et al*., 2013; Mehdi *et al*., 2016). The C‐terminal half of the protein sequence shows homology to RXT3, a functionally uncharacterised member of the large RPD3 HDAC complex in yeast, whereas the N‐terminal part only occurs in plant proteins. HDC1 is an intrinsically disordered protein which can interact with multiple proteins including histone deacetylases HDA6 and HDA19, co‐repressor SIN3 ASSOCIATED POLYPEPTIDE 18 (SAP18; Song & Galbraith, 2006), histone‐3 binding proteins INHIBITOR OF GROWTH 2 (ING2; Lee *et al*., 2009), MULTICOPY SUPPRESSOR OF IRA1 (MSI1; Köhler *et al*., 2003) and SHORT LIFE (SHL or SHL1; Müssig *et al*., 2000; Qian *et al*., 2018) as well as histone 1, including all three variants, H1.1, H1.2 and the stress‐inducible H1.3 (Wierzbicki & Jerzmanowski, 2005; Rutowicz *et al*., 2015). A truncated RXT3‐like version of HDC1, missing the N‐terminal part, showed diminished interaction with the histone deacetylases but still strongly interacted with H1 (Perrella *et al*., 2016). Knockout of HDC1 causes hyperacetylation of H3 lysines 9 and 14 (H3K9K14) and increases the transcript levels of several genes (Perrella *et al*., 2013). The combined evidence suggests that HDC1 provides a scaffold for protein interactions in HDAC complexes, thereby increasing their stability and activity. Unlike HDACs, HDC1 is a constitutively expressed, ubiquitous, single‐copy gene in *A. thaliana* (and other diploid plant species). Experimentally modifying its expression has quantitative effects, suggesting that it is an essential and rate‐limiting part of HDAC complexes. *Hdc1* knockout plants have smaller leaves and shorter petioles and delayed flowering compared to wildtype (WT), while overexpressors have the opposite phenotypes. Notably, knockout/overexpression of *HDC1* increases/decreases the sensitivity of plants to the stress hormone abscisic acid (ABA) and to salt. The hyposensitive overexpressing lines showed increased growth and yield during moderate water limitation, which could reflect a possible advantage of lower stress sensitivity under moderate stress (Perrella *et al*., 2013). In a separate study, HDC1 was found to cause insensitivity to P starvation in roots (Xu *et al*., 2020).

Despite the clear phenotypes, the gene targets and mechanism through which HDC1 regulates stress responsiveness remain to be identified. The aim of this study was to address these open questions using the salt sensitivity of *A. thaliana* seedlings as a controlled and tractable experimental system. Through a combination of ‘omics’ and genetics approaches, we identified two stress‐inducible genes that mediate the effect of HDC1 on salt sensitivity, and we discovered that HDC1 attenuates their transcriptional response via a dual mechanism involving both histone deacetylation and histone methylation. H1 is required for the latter but not the former. The combined results shed light on a natural process by which plants moderate stress responses.

## Materials and Methods

### Plant material, growth conditions and phenotyping

*Arabidopsis thaliana* (L.) WT, mutants (*hdc1‐1*, *shl1*, *3h1*, *maf5*, *lea* and *h1.1*) and transgenic lines (*HDC1c* and *hdc1‐1/RXT3*) were in the Columbia (Col‐0) background. *Hdc1‐1*, *shl1*, *maf5*, *lea* and *h1.1* come from NASC (http://arabidopsis.info) GABI‐Kat 054G03, N847008, N668580, N654612 and N654890. The triple H1 mutant (*3h1*) was kindly provided by Andrzej Jerzmanowski and Célia Baroux (Rutowicz *et al*., 2015, 2019). For seed germination assays, chromatin immunoprecipitation (ChIP), transcript and proteins analyses, Arabidopsis seeds were sterilised and imbibed for 2 d at 4°C in the dark. Subsequently, the seeds were sown on 0.8% agar plates containing half‐strength Murashige and Skoog salts with 1% sucrose. For information on seed germination assays, please refer to (Perrella *et al*., 2013). In short, media were supplemented with NaCl (Sigma‐Aldrich) at the concentrations given in the figure legends. Salt sensitivity was scored on day 6 after sowing by counting seedlings that had developed green cotyledons and dividing this count by the total number of seeds sown. This phenotype (% established seedlings) encompasses any inhibition of germination as well as post‐germination arrest. For RNA and chromatin extraction, the seedlings were harvested on day 3 after sowing, and some plates were retained to reliably score the phenotypes on day 6. Each experiment was carried out in at least three biological replicates consisting of independent batches of seedlings sown and grown on separately prepared plates. Details on the number of seedlings used per biological replicate are provided in the figure legends.

### PCR

Total genomic DNA was extracted according to (Edwards *et al*., 1991). All PCR reactions were performed with 0.4 units of Taq polymerase (Thermo Fisher, Rodano, Milan, Italy). Total RNA was extracted using innuPREP Plant RNA Kit (Analytik‐Jena, Jena, Germany) or hot phenol method. cDNA was obtained with the Superscript IV kit combined with RNAseOUT (Thermo Fisher) following the manufacturer's procedure. Quantitative RT‐PCR was performed on ABI Prism® 7900HT instrument (Applied Biosystems, Monza, Italy) with Platinum® SYBRGreen® qPCR SuperMix‐UDG with ROX (Thermo Fisher). *C*_t_ values and relative quantifications were analysed as previously described with some modifications: the fold‐changes were expressed in percentage to the control (Fasano *et al*., 2016). Reactions were performed in four technical replicates on three biological replicates. The following cycling conditions were used for quantitative PCR: 2 min at 95°C, 40 cycles of 3 s at 95°C, and 30 s at 59.5°C. Melt curve analysis from 60°C to 90°C was performed to monitor the specificity of the amplification. Primer sequences are reported in Supporting Information Table S1.

### RNA sequencing and data analysis

The sequencing libraries were generated and sequenced in the Glasgow Polyomics Facility (University of Glasgow). Libraries were obtained using Illumina TruSeq stranded mRNA kit by the standard protocols and subsequently sequenced on Illumina NextSeq 500 sequencer to produce single‐end 75 bp long reads.

The raw Fasta files were pre‐processed to trim the 3′ end adapter with Cutadapt (v.1.5; Martin, 2011) and to trim very low‐quality reads with Sickle Software (v.0.940; Joshi & Fass, 2011), allowing for the minimum read length of 54 bp and a quality threshold of 10 (flags ‐q 10, ‐l 54). Transcript expression quantification was performed using Kallisto software (v.0.43.0; Bray *et al*., 2016) against the TAIR10 transcriptome. Read counts related to TAIR10 transcripts were collected, rounded and summarised into gene‐specific read counts. Read statistics are presented in Table S2. DESeq2 software (v.1.24.0; Love *et al*., 2014) was used to identify differentially expressed genes (DEGs for all pairwise comparisons within genotype or condition) and differentially responsive genes (DRGs for condition‐genotype interactions). Genes with a zero read count in all conditions and genotypes were removed before these analyses. *P* values were adjusted for multiple testing (*P*_adj_).

### Chromatin immunoprecipitation

Chromatin immunoprecipitation assays were performed with 2 g of tissue as described previously with minor modifications (Sani *et al*., 2013). A Bioruptor sonicator (B01020001; Diagenode, Seraing (Ougrée), Belgium) was used to shear the chromatin using the following settings: 20 cycles × 30 s ON, 30 s OFF at high power. Anti‐H3K9K14Ac and H3K27me3 antibodies were used to IP the chromatin (Diagenode pAb‐005‐050 and pAb‐069‐050).

### ChIP sequencing and data analysis

Sequencing of the ChIP DNA was carried out in the Glasgow Polyomics Facility (University of Glasgow). A DNA library was prepared using the NEBNext® Ultra™ DNA Prep Kit (New England BioLabs®Inc., Hitchin, UK) according to the manufacturer's protocol, size selected with SPRIselect Beads and amplified by PCR. The libraries were then sequenced with Illumina NextSeq 500 system producing single 75 bp long reads.

The raw Fastq files were pre‐processed using Cutadapt (v.1.9.2; Martin, 2011) and Sickle (v.0.940; flags ‐q 10, ‐l 54) software (Joshi & Fass, 2011) to remove adapters and do the quality‐based filtering, respectively. Reads were then aligned to the *A. thaliana* genome (TAIR10) using Bowtie (v.0.12.7; Langmead *et al*., 2009), allowing for unique alignments only with up to two mismatches in the first 54 bases (*flags ‐m 1*, *‐n 2*, *‐l 54*). The alignment files in SAM/BAM format were then sorted, and the duplicated reads of the same orientation were removed using Samtools (v.0.1.19; Li *et al*., 2009), and the resulting alignment positions were stored in BED files. For each sample, the aligned reads positions were shifted in the 3′ end direction by half of the sample‐specific mean fragment length to represent the centres of sequenced fragments, counted in 200‐bp long windows using Sicer (v.1.03; Zang *et al*., 2009) and resulting profiles stored in bedGraph files, for uploading into genome browsers. Differentially acetylated H3K9K14 regions (DARs; salt/control ≥1.5 fold) in different parts of the TAIR10 genome in HDC1c and *hdc1‐1* were identified with ChipDiff software (Xu *et al*., 2008) using the gap internal length parameter of 400 bp. The selection of other ChipDiff parameters as well as procedures for both the optimisation of the gap internal length and the linking DARs with adjacent genes, were described previously in (Sani *et al*., 2013). Identification of enriched motifs was obtained using Homer (http://homer.ucsd.edu/homer/).

### ChIP‐PCRs

ChIP‐qPCR was performed at the following cycles: 95°C × 3 min, 95°C × 3 s, 59.5°C × 30 s (40 cycles), 95°C × 1 min, and 60°C × 30 s (Melting curve). Reactions were performed on four technical replicates and three independent biological replicates.

Relative enrichment for ChIP‐qPCR assays was calculated, as shown in Kaiserli *et al*. (2015) and Perrella *et al*. (2018). Histone acetylation and methylation enrichment over loci were determined by normalising immunoprecipitated DNA against genomic DNA for the regions highlighted in Fig. 2 (see later) and indicated as percentage of nuclear DNA (% Input). Primer sequences are reported in Table S1. Primer positions are indicated in Fig. 2 (see later).

### Protein extraction and western blot analysis

Arabidopsis seedlings were snap‐frozen in liquid N_2_ on day 3 after sowing. Total protein was extracted by grinding 50 mg of tissue in 4× Laemmli sample buffer (Brown *et al*., 2005) and boiling. SDS/PAGE analysis was performed using 15% acrylamide gels and the Bio‐Rad *Trans*‐Blot Turbo transfer system was used for western blot transfer. After transfer, the membranes were stained with Ponceau solution (SIGMA). The following antibodies were used for western blot analysis: anti‐H3K9K14Ac, anti‐H3K27me3 and anti‐UGPase (Diagenode and Agrisera, Vännäs, Sweden all 1 : 1000 dilution). Detection was performed with the Bio‐Rad ChemiDoc system. Band intensity was quantified using ImageJ.

### Bisulphite sequencing

Genomic DNA was extracted from roots of Col‐0, *hdc1‐1* and *3h1* using InnuPrep Plant DNA Kit (Analytik‐Jena), following the manufacturer's instructions. Three independent experiments were performed, each comprising pooled samples of over 100 seedlings. 150 ng of genomic DNA was treated with bisulphite using the EpiTect Bisulfite kit (cat#59104; Qiagen). The primers listed in Table S1 were used to amplify the *LEA* and *MAF5* region across promoter and TSS sequences. The fragments were then cloned in pCR 2.1 vector using the TOPO TA Cloning Kit (Thermo Fisher), following the manufacturer's instructions, for a total of eight individual clones per genotype and per treatment (control and 100 mM salt). The sequences were aligned using LASERGENE Seqman Ultra, and the methylation rates of individual cytosines were calculated through bisulfite conversion rate in percentage.

### CRISPR‐Cas9 mutagenesis

CRISPR‐Cas9‐based mutagenesis on *HDC1* genomic sequence was attempted using the pKIR1.1 vector (Cat. # 85758; Addgene, Watertown, MA, USA) following the plasmid depositors' instructions (Tsutsui & Higashiyama, 2017) with the following modifications. Two gRNAs were designed using Chopchop v.3 (Labun *et al*., 2019) to target *HDC1*'s first exon. The gRNAs were cloned in a tandem array of tRNA‐gRNA using the pGTR vector (Xie *et al*., 2015). Oligonucleotides used to assemble the plasmid were designed using the web tool of the Voytas' lab (Čermák *et al*., 2017) and are listed in Table S1. Engineered vector pKIR1.1_HDC1 was used to transform *3h1* plants. Transformation with pKIR1.1_FD was used as a control. T1 seeds screening was performed by RFP fluorescence in the seed coat under a Nikon SMZ18 stereomicroscope.

## Results

### HDC1 moderates early transcriptional responses of germinating seedlings to salt

We have previously shown that HDC1 alters the response of germinating *Arabidopsis thaliana* to salt or ABA treatment (Perrella *et al*., 2013). Both treatments inhibit radicle emergence, early root growth and cotyledon development. The combined phenotype can be reliably scored 6 d after sowing as ‘percentage of established seedlings’. The salt/ABA‐induced decrease in the seedling establishment was more pronounced in a loss of function mutant (*hdc1‐1*, hypersensitive) and less severe in HDC1 overexpressing lines (OX‐HDC1, hyposensitive) than WT. Complementation of *hdc1‐1* with full‐length genomic HDC1 under its own promoter (*HDC1c*) restored WT behaviour, whereas expressing only the RXT3‐like part of HDC1 in the knockout background (*RXT3*) partially restored the WT level of ABA/salt‐sensitivity (Perrella *et al*., 2013).

To identify genes underpinning the phenotypes, we performed RNA sequencing on *HDC1c*, *hdc1‐1* and *RXT3* seedlings germinating on plates supplemented with 0 (control) or 100 mM NaCl (salt). From each replicate experiment (independently treated seed batch), we extracted and pooled mRNA from half of the seedlings (*c*. 150) on day 3 and scored the phenotype of the remaining seedlings on day 6 (see Fig. S1A for experimental design). The phenotypes confirmed the previously reported differences between the lines (Fig. S1B), with *hdc1‐1* being hypersensitive and *RXT3* showing intermediate sensitivity towards salt compared to *HDC1c*. The 18 RNA samples (three genotypes × two conditions × three biological replicates) were subjected to next‐generation sequencing (RNA‐seq). *HDC1c* was used here instead of WT to ensure the identical epi‐/genetic background of all genotypes. The sequencing reads statistics are available in Datasets S1, S2 and Table S2.

**Fig. 1:**
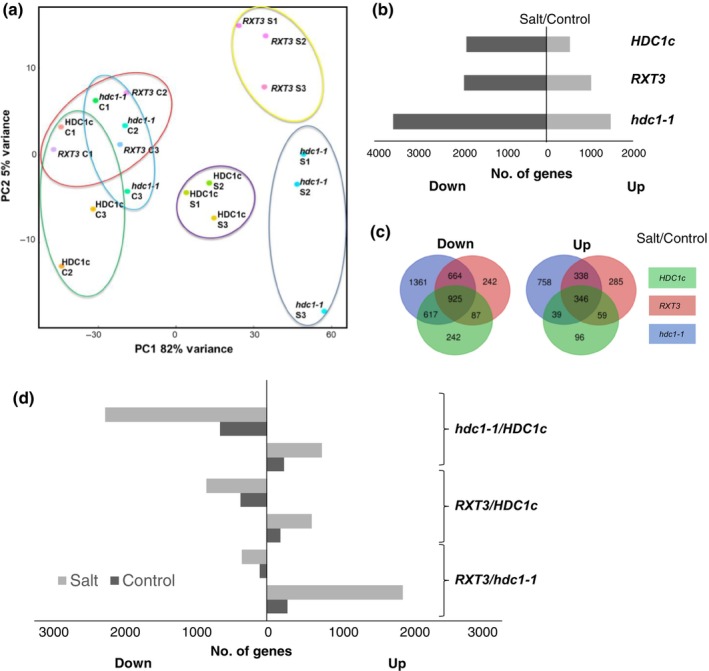
Histone deacetylase complex 1 (HDC1) attenuates the transcriptional responsiveness of *Arabidopsis thaliana* seedlings to salt stress. (a) Principal component analysis (PCA) analysis based on genome‐wide transcript levels in 3 d old seedlings of *hdc1‐1* knockout mutant (*hdc1‐1*), *hdc1‐1* complemented with full‐length HDC1 (*HDC1c*), and *hdc1‐1* expressing the RXT3 motif of HDC1 (*RXT3*). Seeds were germinated on media without (control, C) or with 100 mM NaCl added (salt, S). Each dot represents an independent sample. Biological replicates of the same genotype and condition are circled. For each biological replicate, *c*. 150 seedlings per genotype under each condition were processed. (b) Number of differentially expressed genes (salt/control) in each genotype (fold change ≥ 2, *P*‐value ≤ 0.05). Number of upregulated genes are shown on the right, number of downregulated genes are shown on the left side of the *y*‐axis. (c) Venn diagrams depicting the number of common and unique differentially expressed genes (salt/control) between *HDC1c*, *RXT3* and *hdc1‐1*. (d) Number of genes differentially expressed between genotypes grown on control or salt media.

Normalised mRNA levels, in the form of Kallisto‐generated TPM values, for all genes in each sample are provided as Dataset S1. Principal component analysis (PCA) of the data (Fig. 1a) placed the replicate samples obtained from independent experiments closely together and clearly distinguished between control (C) and salt‐treated (S) samples (PC1 explaining 82% of variance). The different genotypes grouped together in control conditions but separated in the salt treatment. Salt‐treated *hdc1‐1* and *RXT3* were separated from salt‐treated *HDC1c* (primarily by PC1) and from each other (primarily by PC2, explaining 5% of the variance). The analysis indicated that the three lines had similar transcriptomes in control conditions but differed in their transcriptional response to salt. To quantitatively compare salt‐responsiveness between the genotypes we calculated the salt/control ratio of mRNA levels for each gene and identified DEGs applying a cut‐off of at least 2‐fold change with a *P*‐value ≤ 0.05, adjusted for multiple testing (Dataset S2). In *HDC1c*, 1871 genes were downregulated, and 540 genes were upregulated by salt (Figs 1b, S2A). A considerably higher number of DEGs (3567 downregulated and 1481 upregulated) was identified for *hdc1‐1* and an intermediate number (1919 downregulated and 1029 upregulated) for *RXT3* (Figs 1b, S3A, S4A). Venn diagrams show that most of the salt/control DEGs in *HDC1c* were also differentially expressed in the mutant lines (Fig. 1c). Direct comparison of transcript levels between genotypes (Fig. 1d) identified more DEGs in the *hdc1‐1*/HDC1c comparison than for *RXT3*/*HDC1c* or *hdc1*/*RXT3*. Notably, the number of DEGs between genotypes was always higher in the salt‐treated samples than in the control. Enrichment analysis of functional annotations of salt‐regulated genes based on GO‐terms and keywords using David (Huang *et al*., 2009) revealed similar terms in all genotypes (Figs S2B–S4B). Annotations related to metabolism and stress response were most enriched among downregulated genes, and annotations related to transcription and metabolism were most enriched among upregulated genes. Gene ontology analysis of DEGs between genotypes indicated no significant enrichment in control conditions (Figs S5A,B–S7A,B), while annotations of metabolism, stress response, photosynthesis and transcription were enriched when comparing salt‐treated genotypes (Figs S5C,D–S7C,D). The combined results indicate that the genotypes have a similar biological response to salt but differ in the strength of the response and, accordingly in the number of DEGs at a given cut‐off. The transcriptomes of the genotypes recorded on day 3 already reflected the phenotypic differences measured on day 6 with a stronger transcriptional response (hypersensitivity) in the *hdc1‐1* knockout mutant compared to the fully complemented line (*HDC1c*) and an intermediate response in the *RXT3* line. However, the finding that increased responses in the mutants concerned both up‐ and downregulated genes was surprising given the role of histone deacetylation in gene repression. A multi‐factorial DSeq analysis testing the effect of genotype on the transcriptional response to salt further supported this finding (Dataset S2). For example, at *P*_adj_ < 0.001, 201 genes showed a significantly different salt response in *hdc1‐1* compared to *HDC1c*, but the majority (83%) of the DRGs were downregulated in *hdc1‐1* (Fig. S7E). Many DRGs were already stress‐responsive in *HDC1c*, but exhibited a stronger response in *hdc1‐1*, with 79 genes showing stronger downregulation and only 11 genes showing stronger up‐regulation (*P*_adj_ < 0.001; Fig. S7F). These response profiles reflect again increased stress sensitivity of *hdc1‐1* but cannot be directly linked with increased histone acetylation in the mutant. A likely explanation is that a small number of direct HDC1‐targets tune the sensitivity of stress perception in the seedlings, while the large majority of the observed transcriptional differences are a consequence rather than the cause of enhanced stress sensitivity in the mutant.

### HDC1 moderates salt‐induced H3K9/14 hyperacetylation and transcriptional activation of stress‐induced genes

To identify potential direct targets of HDC1, we isolated nuclei from control and salt‐treated *HDC1c* and *hdc1‐1* seedlings and performed chromatin immunoprecipitation (ChIP) with an antibody against acetylated lysines 9 and 14 in histone 3 (anti‐H3K9K14Ac). ChIP samples from the first experiment were subjected to Illumina sequencing (ChIP‐seq). The sequencing reads statistics and analysis of the ChIP‐seq experiment are provided as Dataset S3 and Table S3; Figs S8, S9. ChIP samples from three additional experiments (biological replicates) were then analysed by ChIP‐qPCR for the selected candidate genes. To pinpoint possible candidates for a causal link between HDC1 function and salt sensitivity, we started from loci with increased H3K9K14Ac in salt‐treated *hdc1‐1* (198 genes; Dataset S3). Further, we examined (1) the position and distribution of the acetylation mark, (2) the expression pattern (RNA‐seq dataset), (3) publicly available information on tissue expression (e‐FP browser; Winter *et al*., 2007) and (4) any other published information. Based on the combined evidence, four genes were chosen for the subsequent experiments. *LATE EMBRYOGENESIS‐ABUNDANT* (*LEA*, AT2G21490), a member of a large gene family encoding early desiccation‐protective proteins, was previously reported to be induced during seed development, germination (Candat *et al*., 2014) and in response to water and cold stress (Miura & Tada, 2014). According to published transcriptome data collated in the eFP browser, it is mostly expressed in dry seeds and siliques (Fig. S10). *MADS AFFECTING FLOWERING 5* (*MAF5*, At5g65080) is a negative regulator of flowering time in short days (Ratcliffe *et al*., 2003; Fujiwara *et al*., 2010); however, the eFP browser indicates high expression in seeds suggesting additional functions in seed germination (Fig. S10). *ABSCISIC ACID INSENSITIVE 3* (*ABI3*, At3g24650) encodes a transcription factor required for seed maturation, development (Kurup *et al*., 2000) and pigment regulation by ABA. *Abi3* mutants have reduced sensitivity to ABA during germination (Holdsworth *et al*., 2008). *RAB GTPASE HOMOLOG B18* (*RAB18*, At5g66400) is known to be induced by ABA and salt (Jeannette *et al*., 1999), and the eFP browser indicates preferential expression in seeds (Fig. S10).

**Fig. 2:**
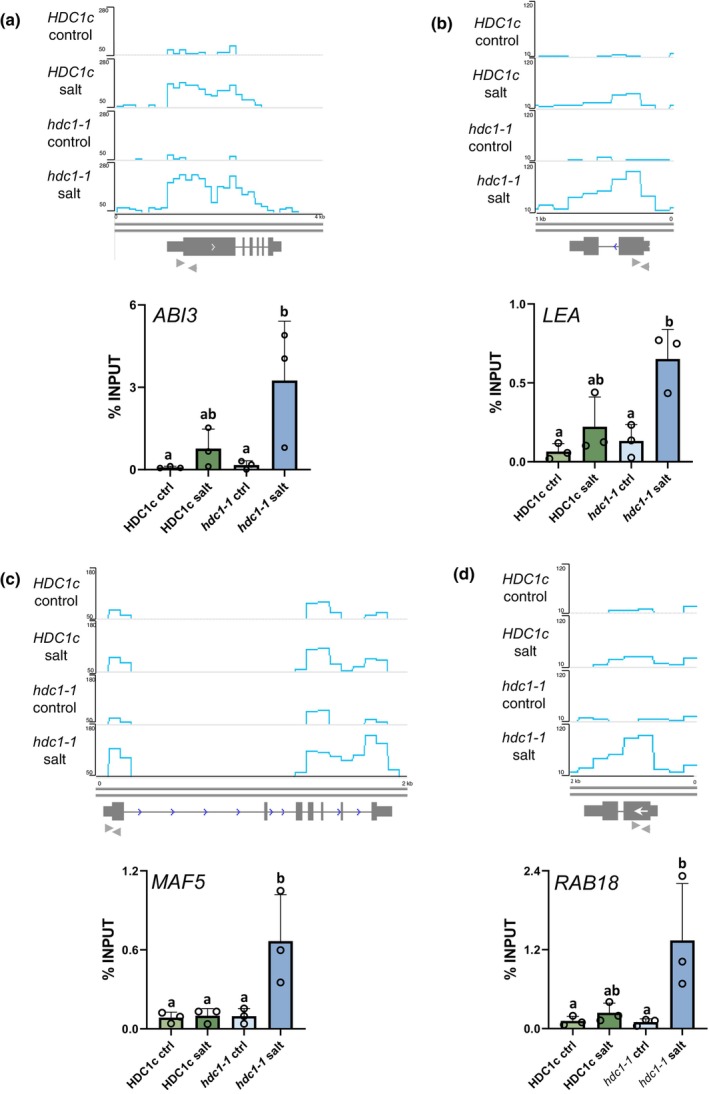
Histone deacetylase complex 1 (HDC1) attenuates histone acetylation of *ABSCISIC ACID INSENSITIVE 3 (ABI3)*, *LATE EMBRYOGENESIS‐ABUNDANT (LEA)*, *MADS AFFECTING FLOWERING 5* (*MAF5)* and *RAB GTPASE HOMOLOG B18* (*RAB18)* in salt‐treated *Arabidopsis thaliana* seedlings. H3K9K14Ac coverage on four genes in *HDC1c* and *hdc1‐1* seedlings on control or salt media. (a) *ABI3* (At3g24650), (b) *LEA* (At2g21490), (c) *MAF5* (At5g65080), (d) *RAB18* (At5g66400). The chromatin immunoprecipitation (ChIP)‐seq profiles at the top of each figure show normalised read numbers over 200‐bp windows. Genes with 3′/5′ UTRs are represented as boxes, white arrowheads indicate the direction of transcription, and grey arrowheads indicate the position of primers used for ChIP‐qPCR. The graphs at the bottom of each figure plot amount of anti‐H3K9K14Ac ChIP DNA determined by qPCR in % of Input. Bars are means of three independent biological replicates (reported above the bars) ± SD. Letters indicate differences at *P* < 0.05 (one‐way ANOVA). For ChIP experiments *c*. 3000 seedlings per genotype under control and salt conditions were processed for a total of four independently grown batches. One biological replicate was subjected to sequencing, while the others were analysed by ChIP‐qPCR. Plant material was obtained from 3 d old *HDC1c* and *hdc1‐1* seedlings.

**Fig. 3:**
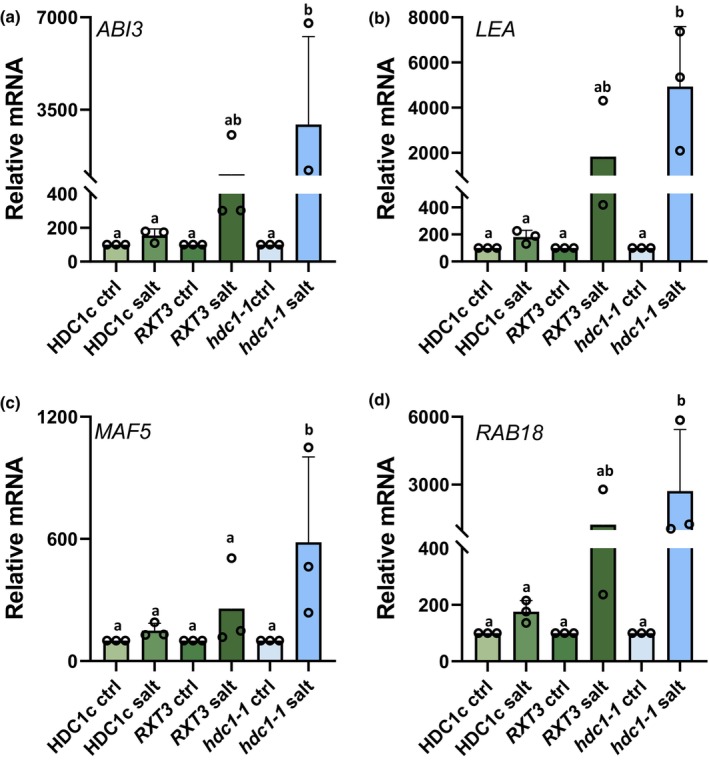
Histone deacetylase complex 1 (HDC1) attenuates transcriptional induction of *ABI3*, *LEA*, *MAF5* and *RAB18*  in salt‐treated *Arabidopsis thaliana* seedlings. mRNA levels of *ABI3* (a), *LEA* (b), *MAF5* (c) and *RAB18* (d) in 3 d old seedlings of *HDC1c*, *RXT3* and *hdc1‐1* grown on control or salt media determined by qPCR and normalised to the housekeeping gene SufE/NifU (*ISU1)* (AT4G22220). Bars are means of three independent biological replicates (reported above the bars) ± SD. Different letters indicate differences at *P* < 0.05 (one‐way ANOVA). For each biological replicate, *c*. 150 seedlings per genotype under each condition were processed.

Fig. 2 shows H3K9K14 acetylation levels associated with the four genes as obtained by ChIP‐seq (upper panel) and by ChIP‐qPCR (lower panel). All four genes showed H3K9K14 hyperacetylation in *hdc1‐1* compared to *HDC1c* upon salt. Additional RT‐qPCR experiments showed up‐regulation by salt leading to significantly higher transcript levels in salt‐treated *hdc1‐1* than in salt‐treated *HDC1c* and intermediate levels in *RXT3* (Fig. 3). It is important to note that the effect of HDC1 on these genes was salt‐conditional since neither transcript nor H3K9K14Ac levels differed between the genotypes under control conditions. In summary, salt treatment increases histone acetylation and transcript levels of the four genes, and both responses are moderated by HDC1.

### *LEA* and *MAF5* mediate salt‐inhibition of seed germination downstream of *HDC1*

**Fig. 4:**
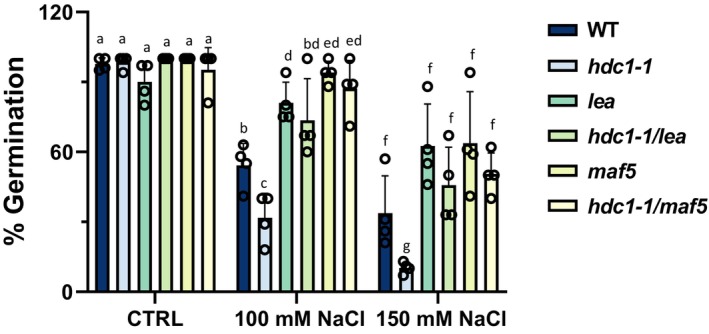
Histone deacetylase complex 1 (HDC1) regulates seed germination in *Arabidopsis thaliana* via *LEA* and *MAF5*. Germination of wild type (WT), knockout mutants *hdc1‐1*, *lea*, *maf5* and double knockout mutants *hdc1‐1/lea* and *hdc1‐1/maf5*. Percentage of established seedlings was scored in independent experiments with ≥ 50 seedlings per experiment on day 6 after sowing on control media and media supplemented with 100 or 150 mM NaCl. Bars are means of four independent biological replicate experiments ± SD (reported above the bars). Different letters indicate significant differences (*P* < 0.05; one‐way ANOVA) between genotypes and compared to control.

For *LEA* and *MAF5*, we were able to obtain homozygous knockout mutants in Col‐0 background and generate double mutants with *hdc1‐1*. In control conditions, seeds of WT, *hdc1‐1*, *lea*, *maf5* and double mutants *hdc1‐1/lea*, *hdc1‐1/maf5* germinated similarly well (Fig. 4). With increasing concentration of NaCl, all lines displayed decreasing germination rates. However, while *hdc1‐1* was significantly more sensitive to the treatments than WT, *lea* and *maf5* were less sensitive (Fig. 4). In all conditions, the germination rates of the *hdc1‐1/lea* and *hdc1‐1/maf5* double mutants were similar to those of *lea* and *maf5* single mutants. Thus, the knockout of *LEA* or *MAF5* suppressed the salt‐hypersensitive phenotype of *hdc1‐1*. The results show that inhibition of germination by salt is at least partially mediated by *LEA* and *MAF5*, which act downstream of *HDC1*. The exact function of *LEA* and *MAF5* in the salt‐sensitivity of Arabidopsis seedlings remains to be elucidated in the future; here, we focussed on the question of how HDC1 regulates these genes.

### Histone 1 moderates salt‐sensitivity of seed germination and transcriptional responses

**Fig. 5:**
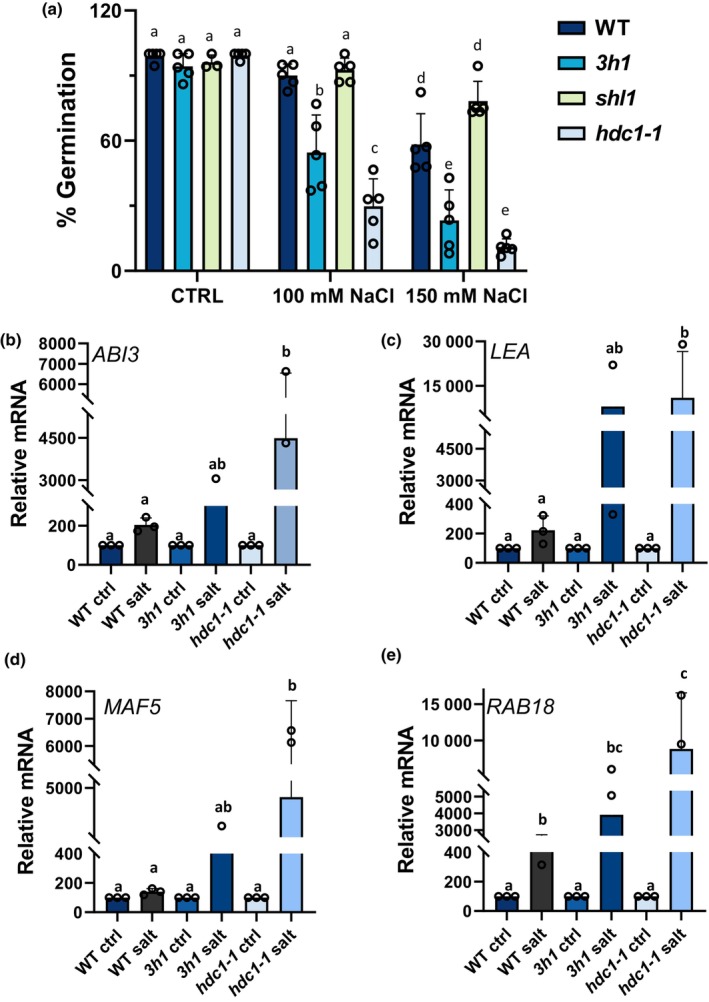
Histone 1 attenuates inhibition of germination and transcriptional induction of *ABI3*, *LEA*, *MAF5* and *RAB18* in salt‐treated *Arabidopsis thaliana* seedlings: (a) Germination rate of wild type (WT), knockout mutants of histone deacetylase complex 1 (HDC1) and histone‐binding protein SHORT LIFE 1 (SHL1) (*hdc‐1‐1* and *shl1*) and triple knockout mutant of histone‐1 variants (*3h1*). Percentage of established seedlings was scored in independent experiments with ≥ 50 seedlings per experiment on day 6 after sowing on control media and media supplemented with 100 or 150 mM NaCl. Bars are means of *n* = 5 independent biological replicates (reported above the bars) ± SD. Different letters indicate significant differences (*P* < 0.05; one‐way ANOVA) between genotypes and compared to control. (b–e) mRNA levels of *ABI3* (b), *LEA* (c), *MAF5* (d) and *RAB18* (e) in 3 d old seedlings of wild type, *hdc‐1‐1* and *3h1* grown on control or salt media determined by qPCR and normalised to the housekeeping gene ISU1. Bars are means three independent biological replicates (reported above the bars) ± SD. Different letters indicate differences at *P* < 0.05 (one‐way ANOVA). For each biological replicate, *c*. 150 seedlings per genotype under each condition were processed.

We have previously shown that HDC1 can interact with many different components of histone deacetylation complexes (Perrella *et al*., 2016). We also reported that the truncation of HDC1 protein to the yeast RXT3‐like (RXT3) region weakened the interaction with HDA6, HDA19 and SAP18 but did not affect the interaction with histone‐binding protein SHL1 or histone 1 variants (H1.1, H1.2 and H1.3). To assess the relevance of HDC1/RXT3‐interacting proteins for stress responses, we obtained *shl1* and triple H1 knockout mutants (*3h1*) and scored seed germination under control and salt conditions (Fig. 5a). All lines displayed similar high germination under control conditions. Salt‐treated *shl1* seeds displayed a similar decrease in germination rate as the WT. By contrast, *3h1* seeds showed a significantly stronger decrease in germination rates than WT, although being less sensitive to salt than *hdc1‐1* (Fig. 5a). Transcript levels of the salt‐induced genes *ABI3*, *LEA*, *MAF5* and *RAB18*, measured by RT‐qPCR, showed a stronger increase upon salt in *3h1* than in WT seedlings but hyper‐activation was less strong than in *hdc1‐1* (Fig. 5b–e). We conclude that SHL1 is not involved in the response of seedlings to salt, whereas H1 attenuates responsiveness albeit to a lesser extent than HDC1.

### H1 is required for *HDC1*‐dependent histone modifications of salt responsive genes

**Fig. 6:**
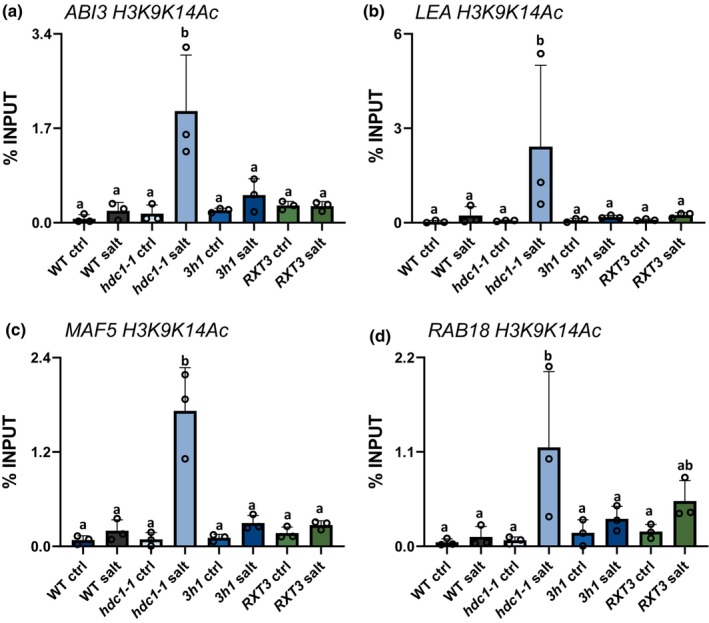
*Arabidopsis thaliana 3h1* mutant seedlings do not mimic the hyperacetylation of stress‐responsive genes observed in *hdc1‐1* mutants. H3K9K14Ac levels (in % of Input) of *ABI3* (a), *LEA* (b), *MAF5* (c) and *RAB18* (d) in wild type, *hdc1‐1* and *3h1*, 3 d old seedlings grown on control or salt media, determined by anti‐H3K9K14Ac‐ChIP‐qPCR using the same primer pairs as in Fig. 2. Bars are means of three independent biological replicates (reported above the bars) ± SD. Letters indicate differences at *P* < 0.05 (one‐way ANOVA). For each ChIP replicate, *c*. 3000 independently grown seedlings per genotype under each condition were processed.

Loss of H1 has been reported to affect DNA methylation as well as specific histone marks including H3K9Ac and H3K27me3 and, to a lesser extent, H3K4me3 (Rutowicz *et al*., 2019). To determine whether loss of H1 alters any of these marks in the selected genes, we immunoprecipitated chromatin from WT, *hdc1‐1*, *RXT3* and *3h1* seedlings, grown on control or salt media for 3 d, using antibodies against H3K9K14Ac or H3K27me3 and performed ChIP‐qPCRs for *ABI3*, *LEA*, *MAF5* and *RAB18*. *3h1* and *RXT3* seedlings did not mimic the strong hyperacetylation of H3K9K14 recorded in *hdc1‐1* seedlings (Fig. 6a–d). This suggests that the HDC1‐mediated deacetylation upon salt does not involve H1.

**Fig. 7:**
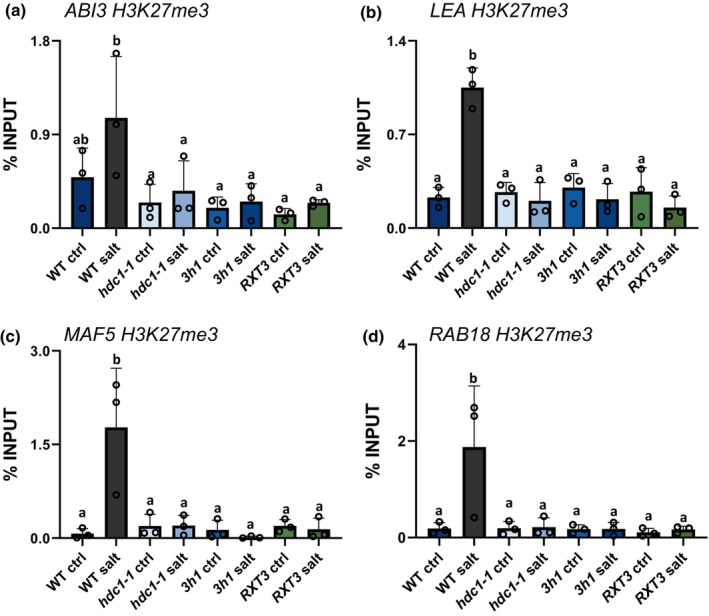
Histone deacetylase complex 1 (HDC1), RXT3 and H1 promote H3K27 tri‐methylation of *ABI3*, *LEA*, *MAF5* and *RAB18* in salt‐treated *Arabidopsis thaliana* seedlings. H3K27me3 levels (in % of Input) of *ABI3* (a), *LEA* (b), *MAF5* (c) and *RAB18* (d) 3‐d old seedlings of wild type, *hdc1‐1*, *RXT3* and *3h1* grown on control or salt media, determined by anti‐H3K27me3‐ChIP‐qPCR using the same primer pairs as in Fig. 2. Bars are means of three independent biological replicates (reported above the bars) ± SD. Letters indicate differences at *P* < 0.05 (one‐way ANOVA). For each ChIP replicate, *c*. 3000 independently grown seedlings per genotype under each condition were processed.

Anti‐H3K27me3 ChIP‐qPCR for the same regions revealed that salt treatment caused a strong increase of H3K27me3 levels in all four genes in WT seedlings. This response was abolished when HDC1 or H1 were non‐functional (Fig. 7a–d). In *hdc1‐1* mutants, H3K27me3 levels increased or decreased only slightly (*ABI3* and *LEA*) or remained the same (*MAF5* and *RAB18*). In *3h1* mutants H3K27me3 levels varied slightly between genes but in no case they showed the strong increase upon salt observed in the WT. The complementation of *hdc1‐1* with the truncated RXT3‐part of HDC1, which binds H1 (Perrella *et al*., 2016), did not restore H3K27me3 to WT levels. Thus, full‐length *HDC1* and H1 are both required for the salt‐induced H3K27me3 hypermethylation of the four genes.

We also monitored total H3K9K14Ac and H3K27me3 levels using western blot analysis in salt‐treated seedlings. The results confirmed H3K9K14 hyperacetylation in *hdc1‐1* (Fig. S11A) and lower H3K27me3 levels in all three mutant lines compared to WT (Fig. S11B).

To interrogate H1‐dependent DNA methylation in the selected genes, we analysed publicly available whole‐genome DNA‐methylomes (Zemach *et al*., 2013) of Col‐0 WT and double *h1.1/h1.2* mutant (named *h1* in Zemach *et al*., 2013). Assessing the number of methylated cytosines revealed very low levels of DNA methylation on *ABI3*, *LEA*, *MAF5* and *RAB18* loci (Fig. S12A). However, a further reduction in *h*1 compared to WT was detected for *LEA* and *MAF5*. In addition, we performed bisulfite sequencing for *LEA* and *MAF5* DNA promoter regions in WT, *hdc1‐1* and *3h1* DNA samples extracted from seedlings grown in control conditions or with 100 mM NaCl. Our results confirmed a very low number of methylated cytosines (15% of all Cs present in the sequence) in control conditions and detected a further reduction in salt‐treated samples. In *hdc1‐1*, the C‐methylation rate was lower than in the WT in control, but no major differences to WT were apparent upon salt treatment (Fig. S12B). *3h1* did not show a decrease in DNA methylation levels. We conclude that *LEA* and *MAF5* are not major targets of the DNA methylation machinery and that the small differences observed are unlikely to have an impact on transcriptional activity.

## Discussion

### HDC1 pulls the brake on stress responses via deacetylation of stress‐inducible genes

Plants respond to environmental stimuli through an intricate network of signalling pathways. While these responses safeguard plant life under challenging conditions, they can have negative impacts on growth and developmental progress. The best solution to this trade‐off will depend on the exact environmental scenario considering strength, duration and frequency of the stress. While enhancing responses could improve plant performance under strong long‐term stress, suppressing unnecessary responses could be beneficial under transient and fluctuating stress conditions. Our research shows that plants have a natural capacity to put a brake on stress responses by generating a chromatin context that hampers the transcription of stress‐induced genes. The potential for much stronger stress responses than naturally displayed is evident in mutants with stress‐hypersensitive phenotypes. For example, a salt (or ABA) hypersensitive seed germination phenotype together with stronger transcriptional responses have been reported for mutants of histone deacetylases and HDAC‐related genes such as HDC1 (Tanaka *et al*., 2008; Chen *et al*., 2010; Chen & Wu, 2010; Perrella *et al*., 2013; Zhou *et al*., 2013; Lee & Seo, 2019; Tu *et al*., 2022) but it was not clear whether the effects were due to a general increase of histone acetylation and transcriptional activity, or whether they were stress‐conditional and gene‐specific. Here we focussed on separating causal from symptomatic effects by monitoring early changes in the transcriptomes and histone acetylomes of salt‐treated seedlings before developmental phenotypes were apparent. The RNA transcriptome suggested that the majority of altered responses in the *hdc1‐1* mutant were the consequences rather than the causes of stronger stress sensitivity because both up‐and downregulation was increased. The histone acetylation data showed that there were hardly any differences between *hdc1‐1* and WT in control conditions, while several hundred loci were differentially acetylated upon stress. These included genes showing a decrease of acetylation in *hdc1‐1*, again indicating that many of the observed differences were downstream of the stress hypersensitivity caused by the mutation. However, there was a clear shift in *hdc1‐1* toward hyperacetylation, reflecting the role of HDC1 in deacetylation. The combined results indicated that HDC1 attenuates stress sensitivity potentially through a small number of genes. This hypothesis was consolidated by the finding that knockout of at least two individual genes, *LEA* or *MAF5*, in *hdc1‐1* background almost completely suppressed the salt‐hypersensitive phenotype of *hdc1‐1*. Thus, releasing the brake by knocking out HDC1 only translates into an enhanced stress response when these two genes are present. Some remaining over‐sensitivity to high salt concentrations in the double mutants indicated that additional genes might also contribute. More *hdc1‐1* double mutants should be generated with mutants for *RAB18*, *ABI3* and other genes in our datasets. All four genes investigated here were hyper‐acetylated (and hyper‐induced) upon stress in *hdc1‐1* mutant but showed no difference in control conditions, suggesting that HDC1‐mediated deacetylation is either itself stress‐induced or requires active transcription of the stress‐induced genes. HDC1 expression is constitutive and, therefore, unlikely to play a role in the former (Perrella *et al*., 2013). RNA Polymerase II (POLII) positioning is a possible mechanism for the latter as histone acetylation increases at nucleosomes with stalled POLII (Martin *et al*., 2021). However, whether it occurs under stress conditions remains to be assessed. Interestingly, we also found that the stress treatment itself led to moderate hyperacetylation in WT. It is therefore possible that stress‐induced hyperacetylation is a prerequisite for the subsequent deacetylation by HDC1, perhaps also on non‐histone proteins (Hartl *et al*., 2017; Narita *et al*., 2019).

### HDC1 and H1 are required for a stress‐induced increase of H3K27me3 – a second brake

We have previously shown that HDC1 interacts with the linker histone H1. A truncated RXT3‐like version of HDC1 is sufficient for this interaction, and *hdc1‐1* plants expressing this version show intermediate phenotypes (Perrella *et al*., 2016). Here we tested the involvement of H1 in the regulation of salt stress‐induced genes with the triple H1 knockout line *3h1* (Rutowicz *et al*., 2015, 2019). *3h1* seedlings were also hypersensitive to salt and showed stronger transcriptional induction of *ABI3*, *LEA*, *MAF5* and *RAB18* by salt than WT, but the changes were less pronounced than in *hdc1‐1*. These findings are consistent with HDC1 acting partially through H1, but this will require proof through genetic analysis by generating a quadruple mutant in the future. Unfortunately, so far, our attempts to obtain such a mutant, either by crossing or by CRISPR‐Cas9 technology, were not successful, indicating possible embryo lethality. This lethality was further supported by aborted seeds in the siliques of the putative quadruple mutant and in the lack of RFP fluorescence in the T1 seeds of *3h1* dipped with HDC1 gRNAs construct (Fig. S13A,B). However, the highest order of homozygous mutant retrieved so far (*h1.2/h1.3/hdc1‐1* triple mutant) mimics *hdc1‐1* with no additive effects suggesting that HDC1 acts downstream of H1 (Fig. S14A–E). Gene editing of H1‐HDC1 interaction sites might be a successful strategy in the future. The next question was how H1 exerts the effect. Previous work showed that loss of H1 alters DNA methylation patterns (Wierzbicki & Jerzmanowski, 2005; Zemach *et al*., 2013; He *et al*., 2019; Choi *et al*., 2020; Liu *et al*., 2021) as well as several histone modifications including H3K9Ac, H3K9me1 and H3K27me3 (Rutowicz *et al*., 2019; Teano *et al*., 2021). The results on bisulfite‐treated samples, particularly on *LEA* and *MAF5*, suggest that DNA methylation is not relevant for HDC1 activity during salt response. However, we cannot exclude that HDC1, together with H1, might exert a function at different plant developmental stages.

Our measurements of H3K9K14Ac levels in the four stress‐responsive genes revealed that *3h1* mutants did not mimic the H3K9K14 hyperacetylation that was apparent in *hdc1‐1* mutants. Therefore, H1 is not required for HDC1‐mediated histone deacetylation. Previously published ChIP‐sequencing data showed that H1 preferentially associates with promoter regions of Polycomb complex PRC2 targets (Teano *et al*., 2021) and immunolocalisation of *3h1* nuclei revealed a reduction of H3K27me3 signal compared to the WT (Rutowicz *et al*., 2019). Our measurement of H3K27me3 levels in the immediate upstream regions of the four genes revealed a strong increase of this histone mark upon salt treatment in the WT, which was lost in both *hdc1‐1* and *3h1* mutants. The finding that H3K27me3, a repressive mark, is deposited on stress‐induced genes upon stress seems counterintuitive but supports the notion of a natural moderation process that prevents overly sensitive responses. Interestingly, *ABI3* is also one of the targets of the PRC2 complex that catalyses the deposition of H3K27me3 during seedlings formation. Such deposition is paramount to avoid the formation of callus‐like structures (Bouyer *et al*., 2011). These results indicate that HDC1 in addition to its role in histone deacetylation also facilitates stress‐conditional deposition of H3K27me3 to target loci. However, WT HDC1 is unable to carry out this function in *3h1* background, and vice versa WT H1 does not mediate the stress‐conditional deposition of H3K27me3 in *hdc1‐1*. The absence of additive effects indicates cooperation, but whether this is due to physical interaction between HDC1 and H1 requires further proof in the future, for example, by identifying H1 binding‐defective HDC1 mutants. Unlike H1‐binding (Perrella *et al*., 2016), the salt‐induced H3K27me3 hypermethylation required the full‐length HDC1 protein, which could indicate that deacetylation is a prerequisite for any function of RXT3‐bound H1 in this process.

**Fig. 8:**
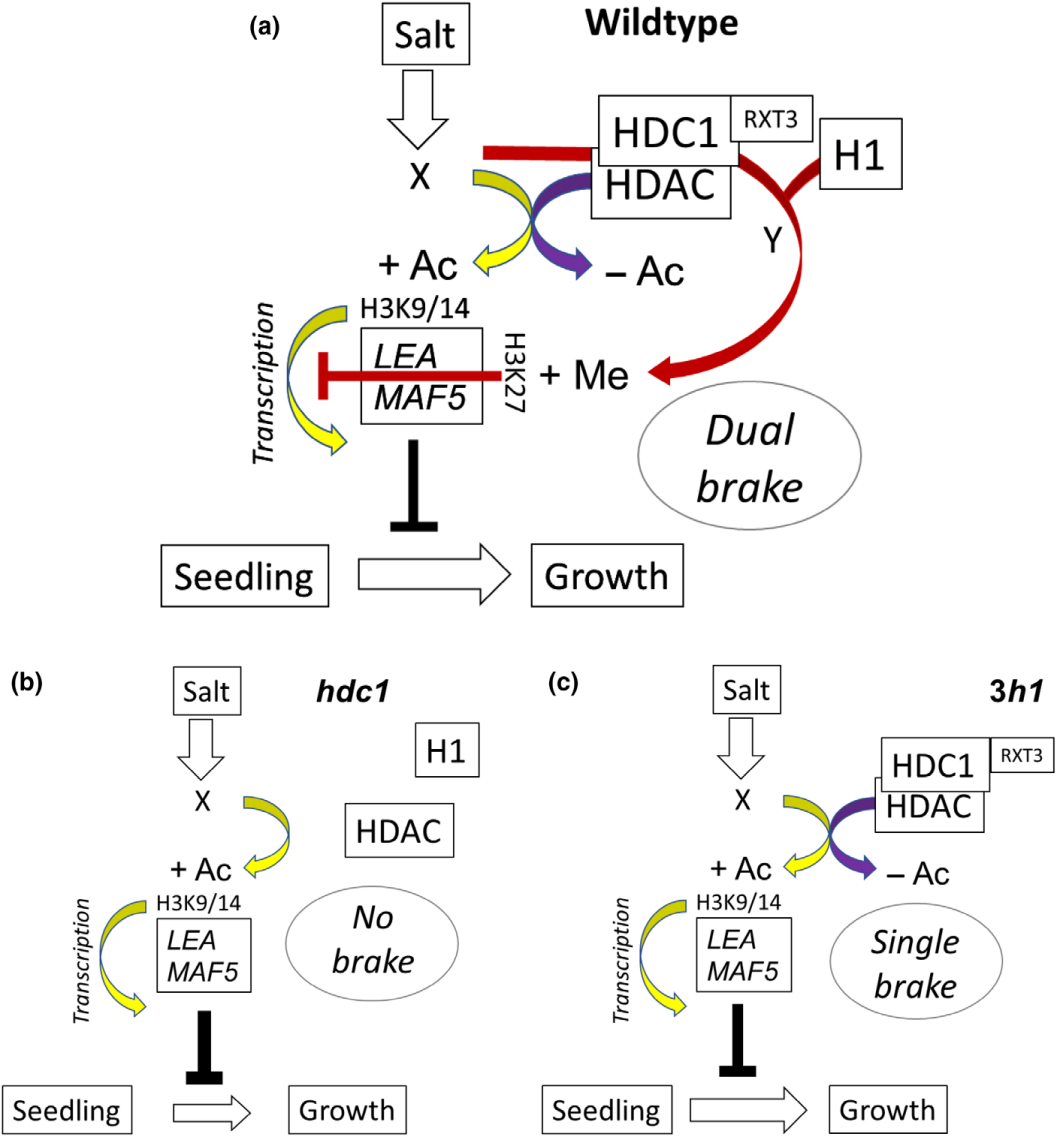
Model of histone deacetylase complex 1 (HDC1) function in fine‐tuning seed growth arrest in response to salt stress. (a) In wild type seedlings, HDC1 stabilises the HDAC complex and mediates H3K9/14 deacetylation of stress‐responsive genes such as *LEA* and *MAF5*, thereby counteracting salt‐induced acetylation and dampening transcriptional up‐regulation. In addition, these genes experience an increase of H3K27me3 upon salt, which requires both HDC1 and H1. This dual brake moderates transcriptional re‐programming and attenuates the inhibitory effect of *LEA* and *MAF5*. (b) Knockout of HDC1 in *hdc1‐1* de‐stabilises the HDAC complex, and both histone deacetylation and histone methylation are compromised. This removes the brake on stress responses. *LEA* and *MAF5* are now hyper‐acetylated and induced by salt causing stronger seed inhibition. (c) Knockout of H1 compromises the salt‐dependent H3K27me3 deposition but not histone deacetylation, and accordingly, the *3h1* mutant seedlings show an intermediate phenotype.

In summary, we found that HDC1 moderates the salt sensitivity of young seedlings through at least two salt‐inducible genes, *LEA* and *MAF5*. We propose that HDC1 dampens their transcriptional upregulation via a dual mechanism (Fig. 8). Firstly, it counteracts salt‐induced histone hyperacetylation through deacetylation. Secondly, it directly represses transcriptional activity via salt‐conditional H3K27 trimethylation. This dual mechanism reinforces the role of HDC1 as a multi‐functional scaffolding protein that enhances the function of HDACs but also enables the recruitment of additional histone‐modifying enzymes. The positioning of LEA and MAF5 downstream of HDC1 is useful to further disentangle causalities within the genetic network underpinning the observed transcriptome‐wide response. The discovery of the HDC1‐(H1)‐LEA/MAF5 regulatory module in *Arabidopsis thaliana* should now be translated to crops to enable optimisation of trade‐offs between stress responsiveness and development for the prevailing environmental conditions in the field.

## Competing interests

None declared.

## Author contributions

GP and AA designed the research. GP, CF, NAD, WF, DM, LC and CC performed the experiments. GP, CF, LD, PH and AA analysed the data. GP and AA wrote the article. GP and CF contributed equally to this work.

## Supporting information


**Dataset S1** Normalised mRNA levels for all genes in each sample by RNA‐seq.

**Dataset S2** Differentially expressed genes salt vs control; multi‐factorial DSeq analysis for genotype vs salt; sequencing reads.

**Dataset S3** Chromatin immunoprecipitation‐seq sequencing reads, statistics and analysis.

**Fig. S1** RNA‐seq experimental setup and *HDC1* expression under salt.
**Fig. S2** Differentially expressed genes and gene ontology *HDC1c* salt/control.
**Fig. S3** Differentially expressed genes and gene ontology *RXT3* salt/control.
**Fig. S4** Differentially expressed genes and gene ontology *hdc1‐1* salt/control.
**Fig. S5** Differentially expressed genes and gene ontology *hdc1‐1/HDC1c*.
**Fig. S6** Differentially expressed genes and gene ontology *RXT3/HDC1c*.
**Fig. S7** Differentially expressed genes and gene ontology *RXT3/hdc1‐1*; DRGs *hdc1‐1/HDC1c*.
**Fig. S8** Differentially acetylated H3K9K14 regions salt vs control and *hdc1‐1/HDC1*; overlaps between RNA‐seq vs chromatin immunoprecipitation‐seq.
**Fig. S9** Gene ontology and promoter motif enrichment of chromatin immunoprecipitation‐seq samples.
**Fig. S10** Spatial expression of *ABI3*, *LEA*, *MAF5* and *RAB18*.
**Fig. S11** Global histone acetylation and methylation levels in Col‐0, *hdc1‐1*, *RXT3* and *3h1*.
**Fig. S12** DNA methylation analyses of H1 and HDC1 knockout lines.
**Fig. S13** Siliques and seeds of *3h1/hdc1‐1*, *h1.1/hdc1‐1* and *3h1* dipped with *HDC1* gRNAs.
**Fig. S14** Germination, RT‐qPCR, chromatin immunoprecipitation‐qPCR and western blot of *h1.2/h1.3/hdc1‐1* triple mutant.
**Table S1** Primers used in this study.

**Table S2** RNA sequencing reads and statistics.

**Table S3** Chromatin immunoprecipitation sequencing reads and statistics.
Please note: Wiley is not responsible for the content or functionality of any Supporting Information supplied by the authors. Any queries (other than missing material) should be directed to the *New Phytologist* Central Office.

## Data Availability

The raw and processed data generated in this study have been deposited in GEO, accession no. GSE206055.
