# Supplementary material for: Histone Deacetylase Complex 1 and histone 1 epigenetically moderate stress responsiveness of Arabidopsis thaliana seedlings
Source: New Phytol. 2023 Aug 10;241(1):166–79. doi: 10.1111/nph.19165 (PMC10953426; doi:10.1111/nph.19165)
Supplement: Supplementary file 4 — Fig. S1 RNA‐seq experimental setup and HDC1 expression under salt. Fig. S2 Differentially expressed genes and gene ontology HDC1c salt/control. Fig. S3 Differentially expressed genes and gene ontology RXT3 salt/control. Fig. S4 Differentially expressed genes and gene ontology hdc1‐1 salt/control. Fig. S5 Differentially expressed genes and gene ontology hdc1‐1/HDC1c. Fig. S6 Differentially expressed genes and gene ontology RXT3/HDC1c. Fig. S7 Differentially expressed genes and gene ontology RXT3/hdc1‐1; DRGs hdc1‐1/HDC1c. Fig. S8 Differentially acetylated H3K9K14 regions salt vs control and hdc1‐1/HDC1; overlaps between RNA‐seq vs chromatin immunoprecipitation‐seq. Fig. S9 Gene ontology and promoter motif enrichment of chromatin immunoprecipitation‐seq samples. Fig. S10 Spatial expression of ABI3, LEA, MAF5 and RAB18. Fig. S11 Global histone acetylation and methylation levels in Col‐0, hdc1‐1, RXT3 and 3h1. Fig. S12 DNA methylation analyses of H1 and HDC1 knockout lines. Fig. S13 Siliques and seeds of 3h1/hdc1‐1, h1.1/hdc1‐1 and 3h1 dipped with HDC1 gRNAs. Fig. S14 Germination, RT‐qPCR, chromatin immunoprecipitation‐qPCR and western blot of h1.2/h1.3/hdc1‐1 triple mutant. Table S1 Primers used in this study. [file NPH-241-166-s001.pdf]

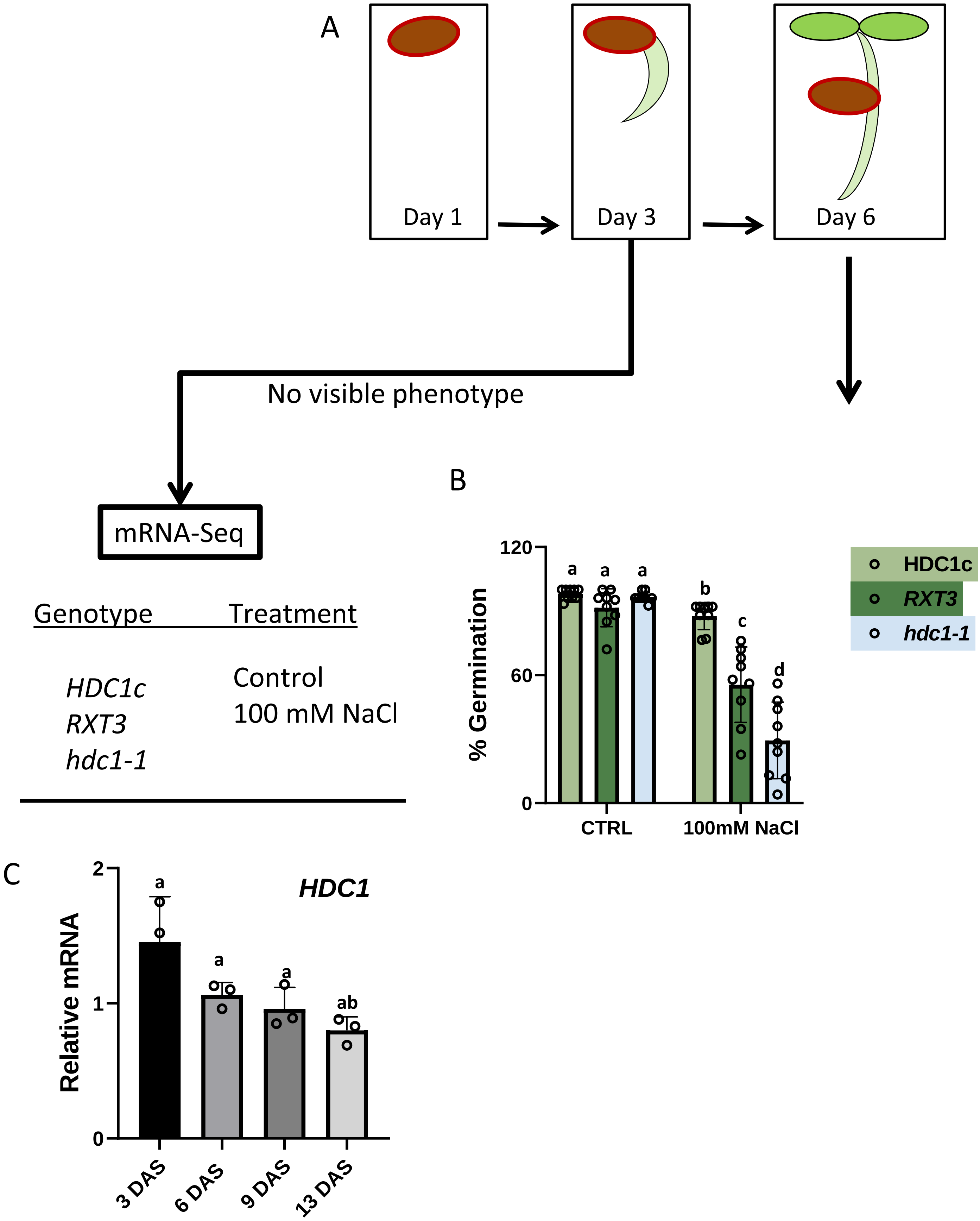

Fig. S1 Schematic representation of the experimental set up for RNA-seq or ChIP-seq sampling. **(A)** Seedlings from *HDC1c* (HDC1 complemented *hdc1-1* mutant), *hdc1-1* mutant and *hdc1-1* mutant over expressing *RXT3* (*RXT3*) motif were harvested at day 3 after sowing. **(B)** At day 6 seedlings for each genotype were scored for germination under control (1/2 MS medium with sucrose) and salt conditions (1/2 MS medium with sucrose supplemented with 100 mM NaCl). Percentage of established seedings was scored in independent experiments with ≥ 50 seedlings per experiment on day 6 after sowing on control media and media supplemented with 100 mM NaCl. (**C**) *HDC1* mRNA levels in wild type seedlings at 3-6-9-13 Days After Sowing (DAS) in 100 mM NaCl. Expression was normalised to those in control conditions. Bars are means of n ≥3 experiments(reported above the bars) ± SD.
For all the experiments seedlings were grown in constant white light (75 mmol m^{-2} s^{-1}). Letters indicate differences at $p < 0.05$ (one-way ANOVA).

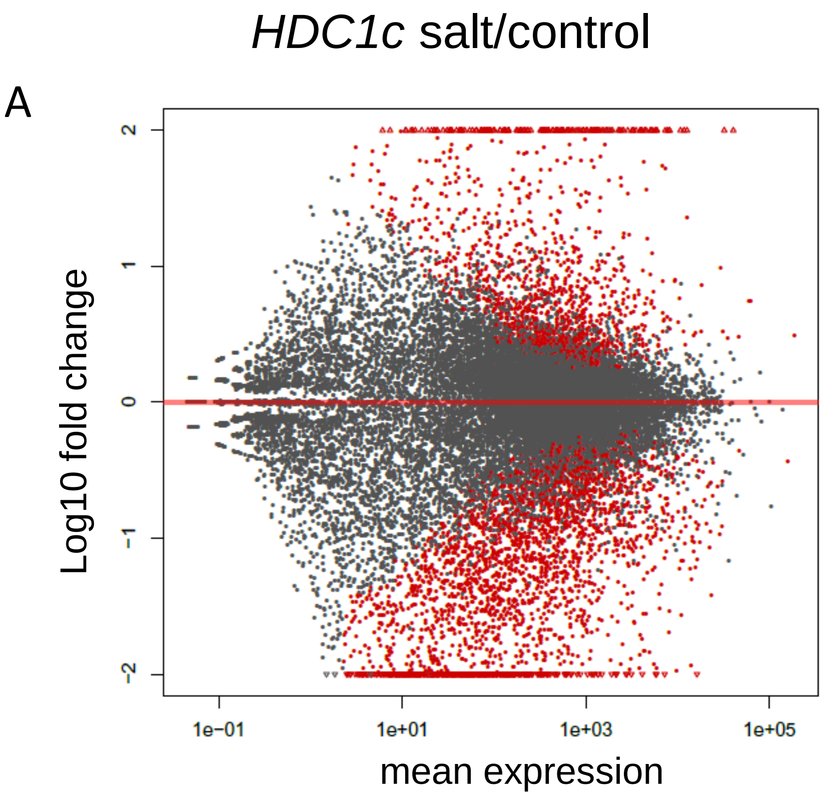

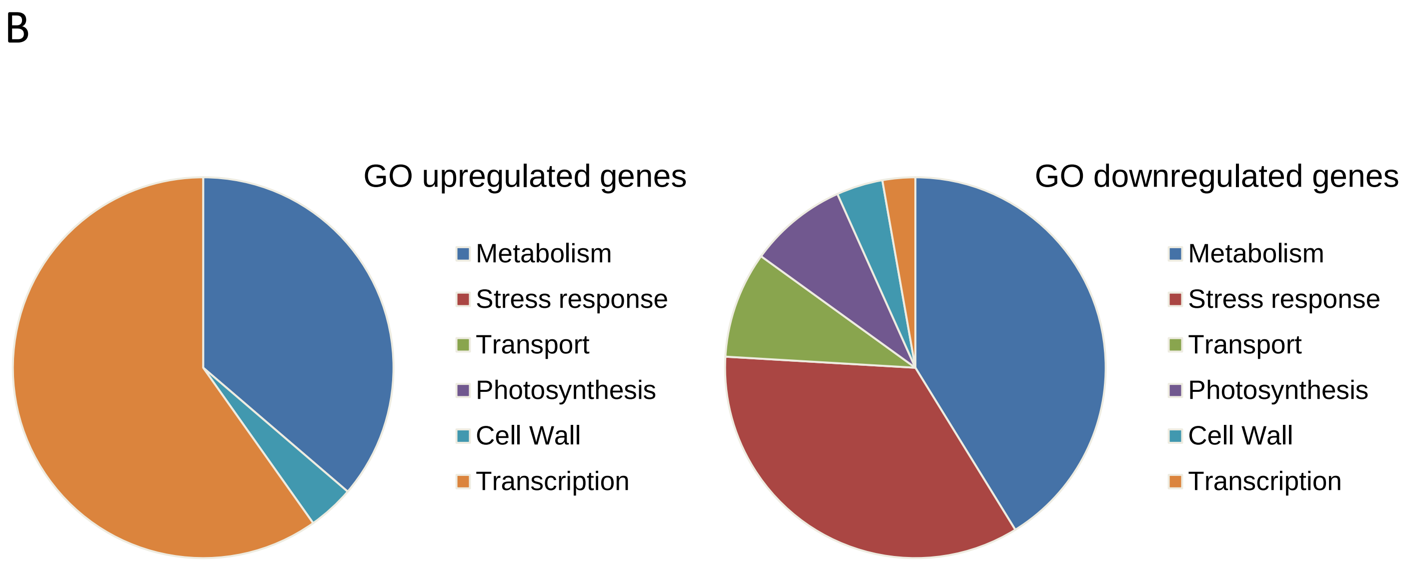

Fig. S2. **(A)** MA plot to visualize differentially expressed genes in the described genotypes and conditions. Red dots indicate genes whose expression changes by log10 and mean average. **(B)** Gene ontology analysis of down and upregulated genes identified by RNAseq with a p-value ≤ 0.05 .

A

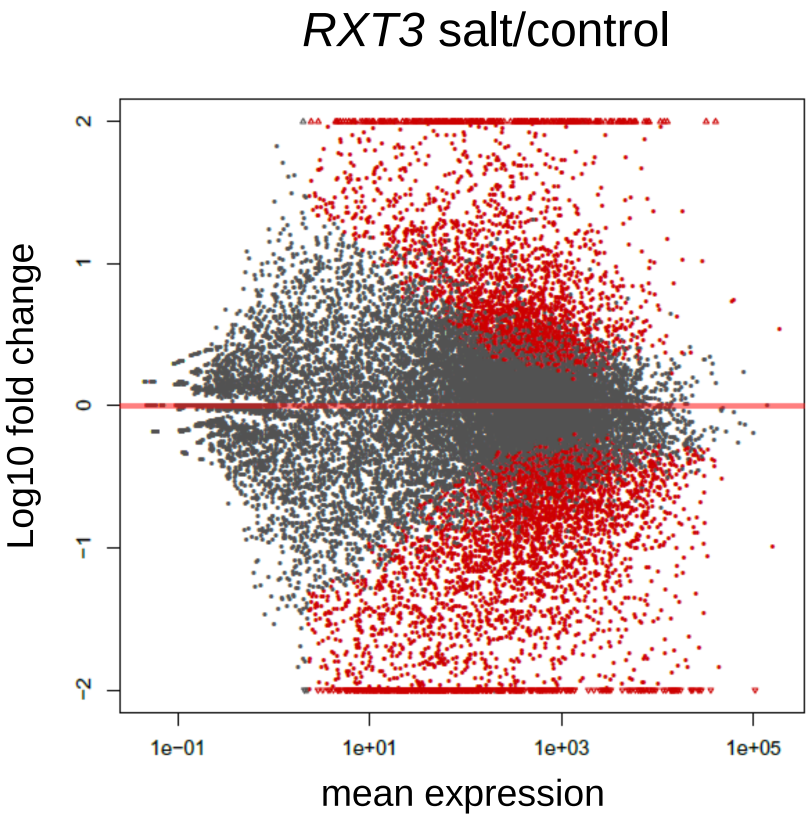

B

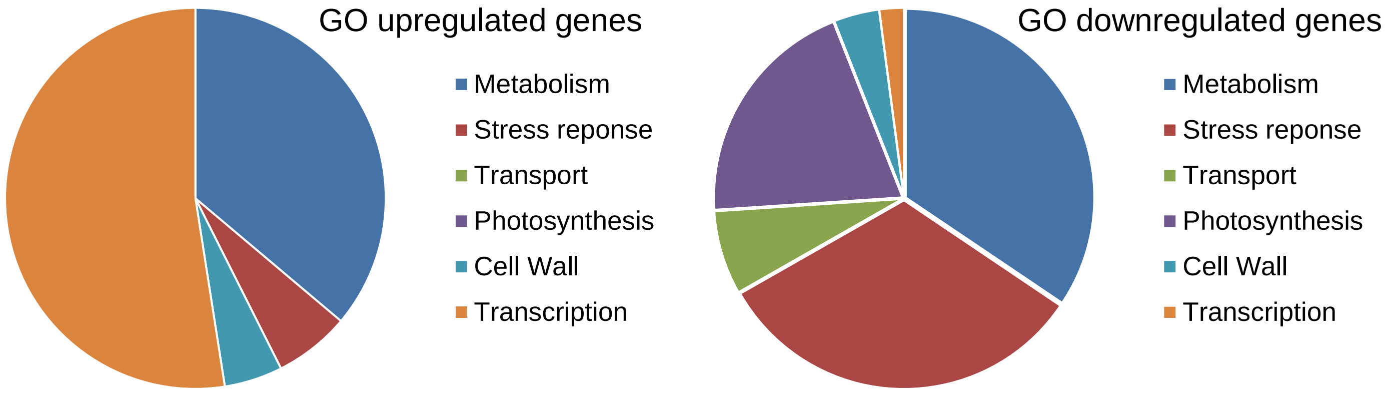

Fig. S3 (A) MA plot to visualize differentially expressed genes in the described genotypes and conditions. Red dots indicate genes whose expression changes by log10 and mean average. **(B)** Gene ontology analysis of down and upregulated genes identified by RNAseq with a p-value ≤ 0.05.

A

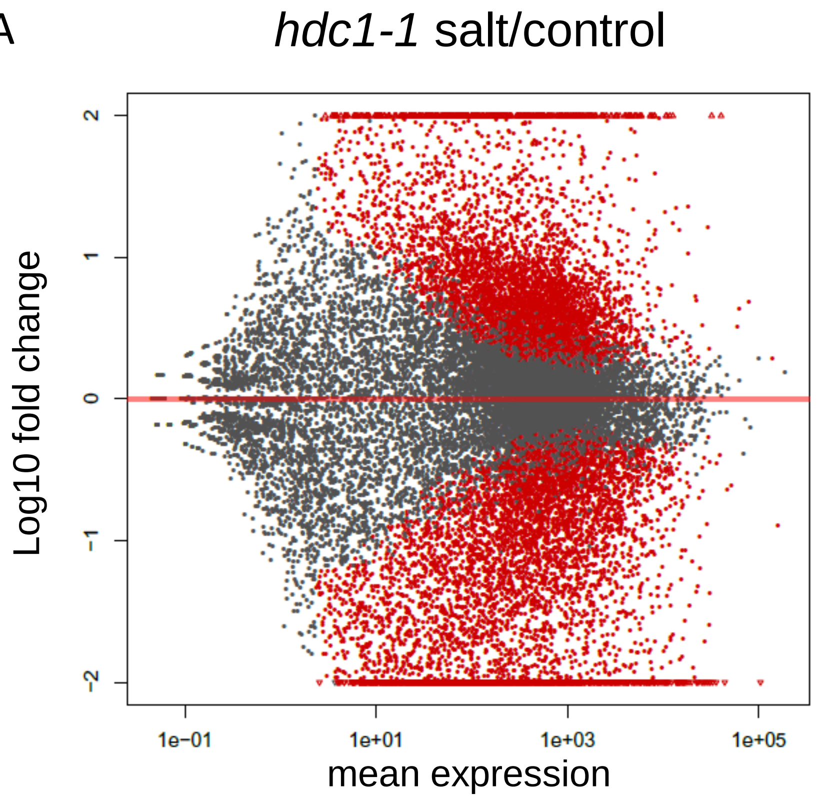

B

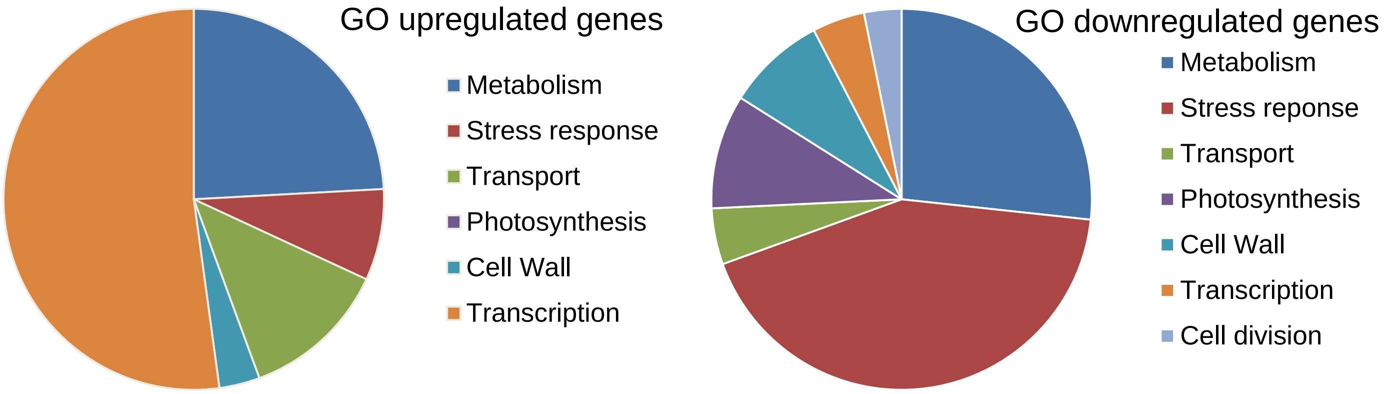

Fig. S4 (A) MA plot to visualize differentially expressed genes in the described genotypes and conditions. Red dots indicate genes whose expression changes by log10 and mean average. **(B)** Gene ontology analysis of down and upregulated genes identified by RNAseq with a p-value ≤ 0.05 .

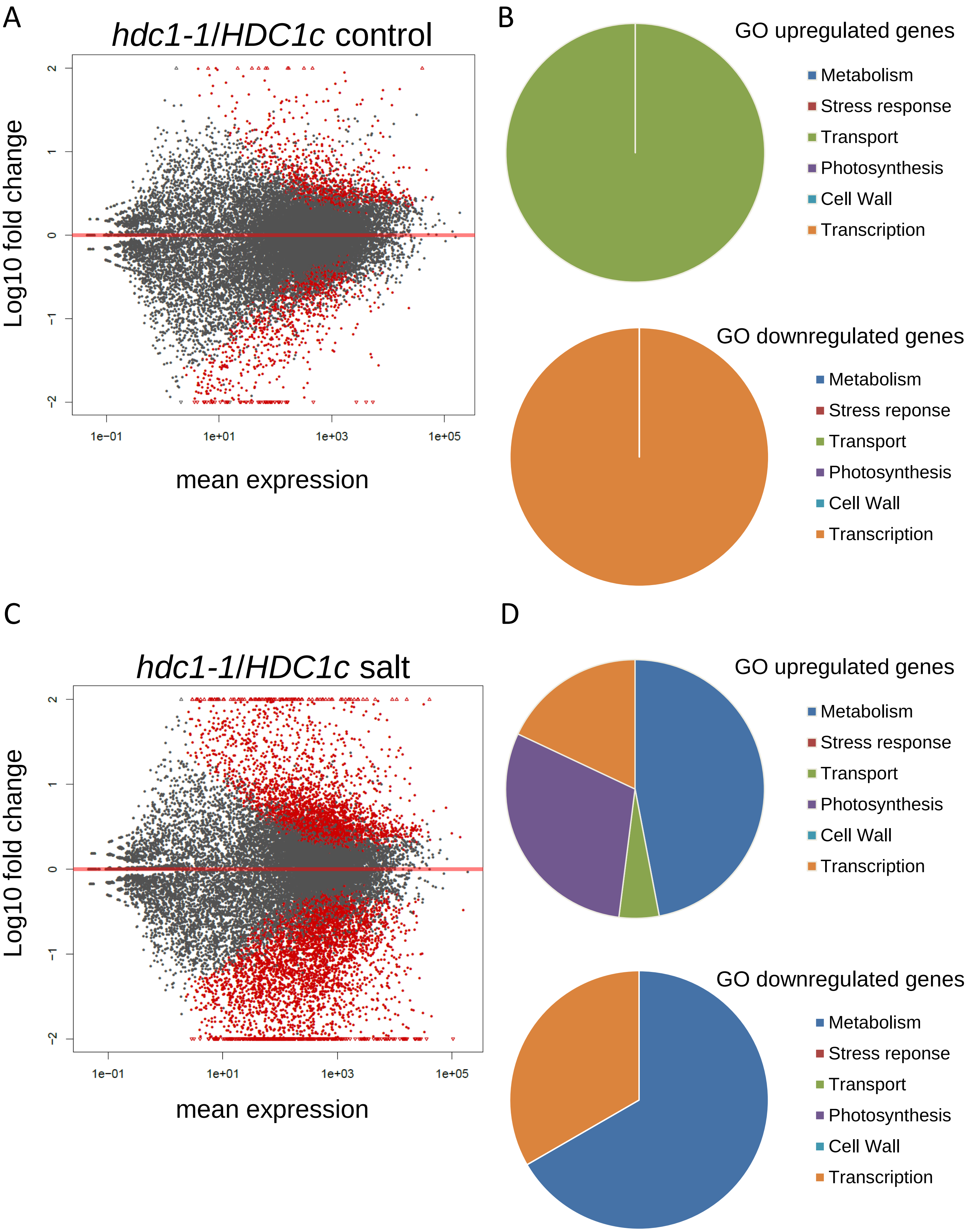

Fig. S5. **(A-C)** MA plot to visualize differentially expressed genes in the described genotypes and conditions. Red dots indicate genes whose expression changes by log10 and mean average. **(B-D)** Gene ontology analysis of down and upregulated genes identified by RNAseq with a p-value ≤ 0.05 .

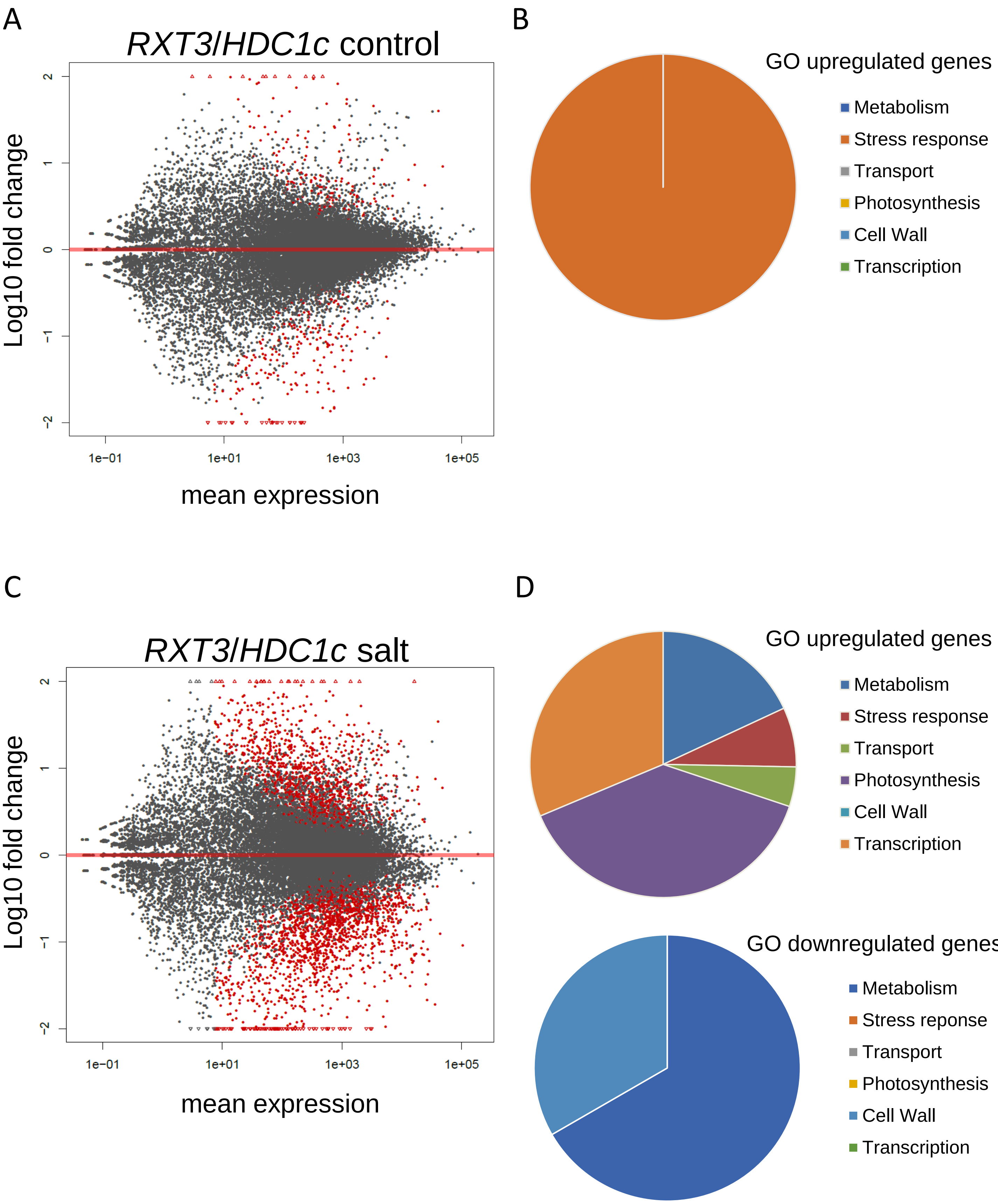

Fig. S6. **(A-C)** MA plot to visualize differentially expressed genes in the described genotypes and conditions. Red dots indicate genes whose expression changes by log10 and mean average. **(B-D)** Gene ontology analysis of down and upregulated genes identified by RNAseq with a p-value ≤ 0.05 .

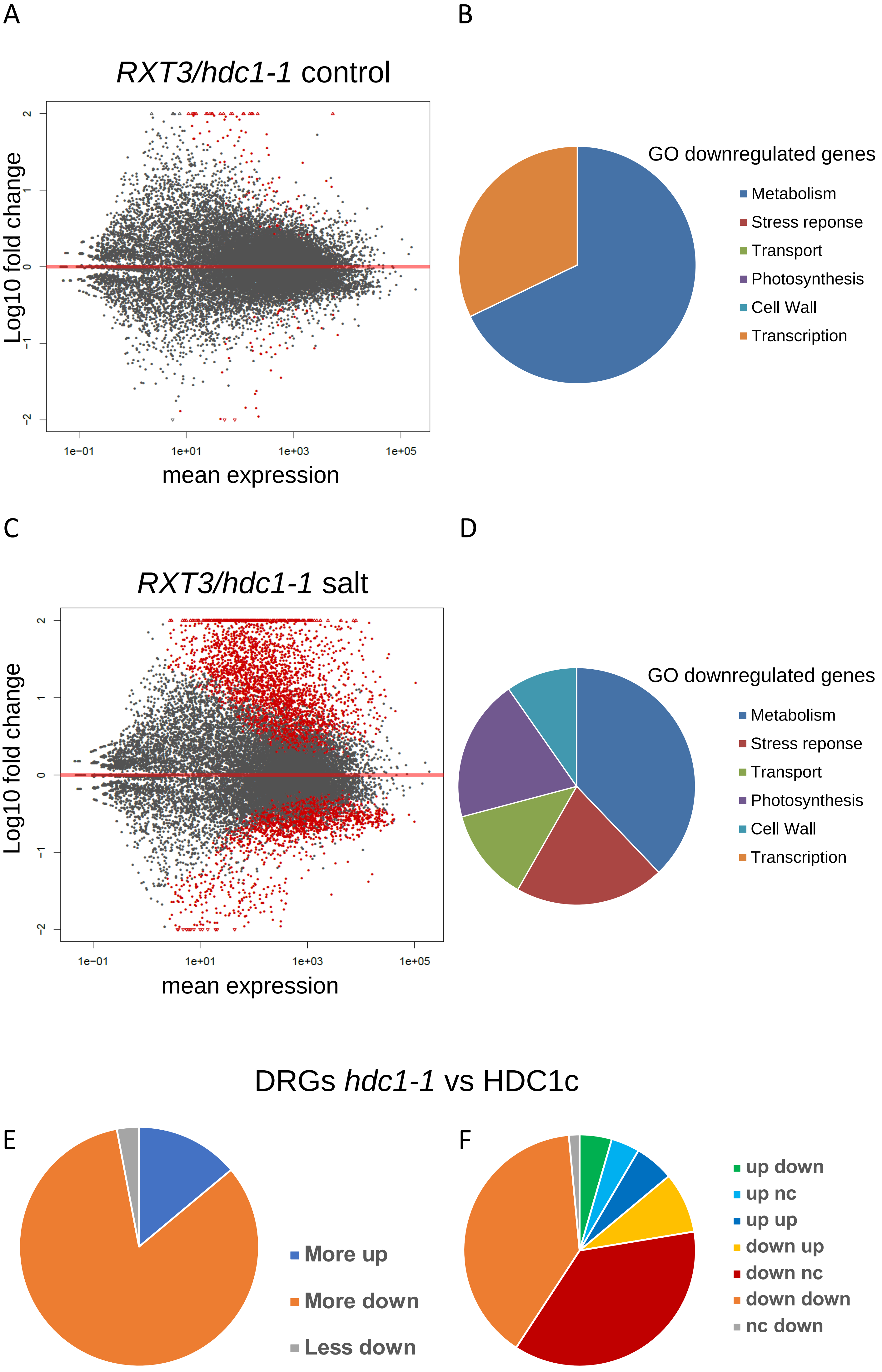

Fig. S7. **(A-C)** MA plot to visualize differentially expressed genes in the described genotypes and conditions. Red dots indicate genes whose expression changes by log10 and mean average. **(B-D)** Gene ontology analysis of down and upregulated genes identified by RNAseq with a p-value ≤ 0,05. **(E-F)** Differential Responsive Genes (DRGs) between *hdc1-1* and *HDC1c* in response to 100 mM NaCl.

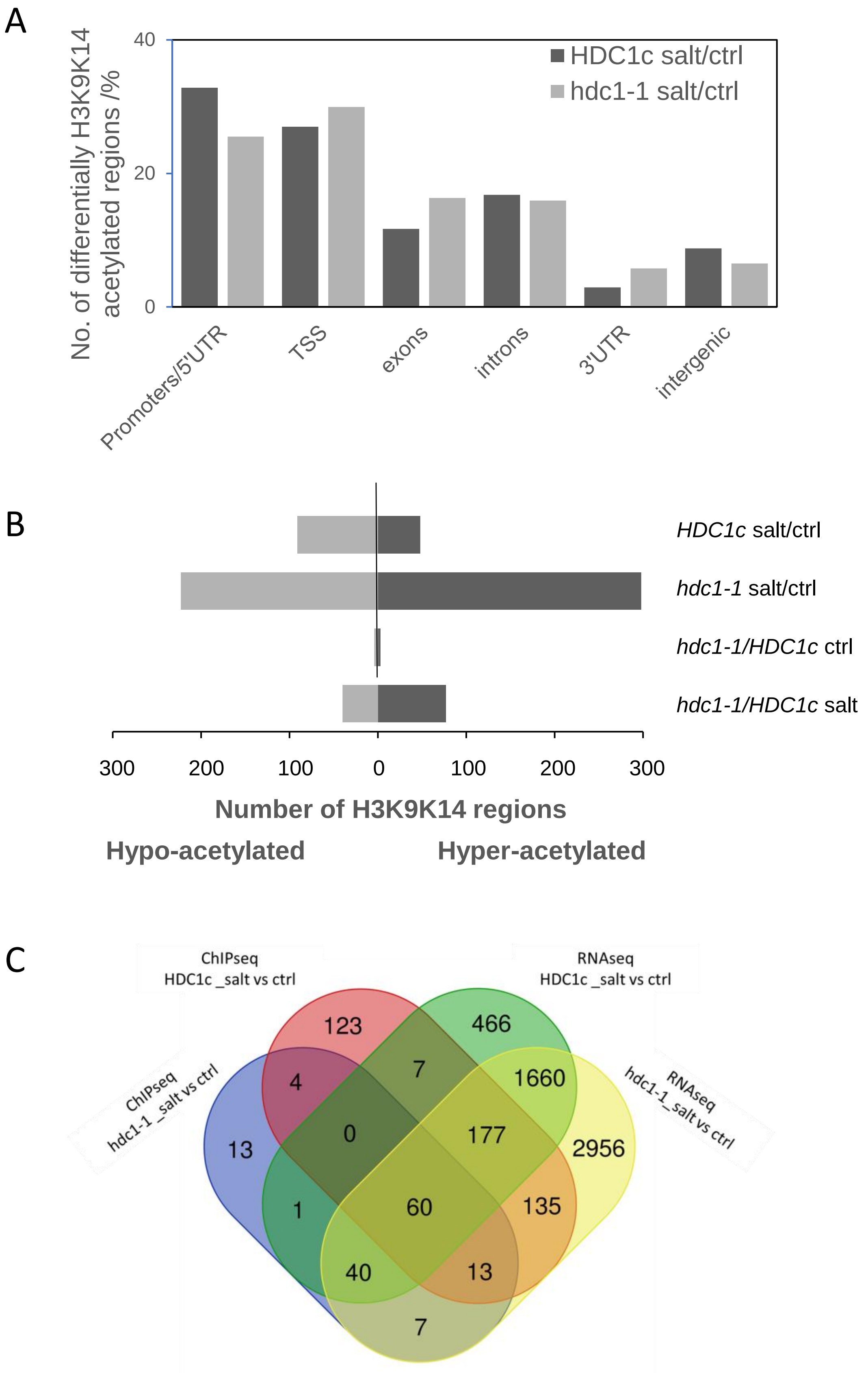

Fig. S8 HDC1 reduces differential histone acetylation in response to salt.
(A) Number of Differentially Acetylated H3K9K14 Regions (DARs) (salt/control ≥1.5 fold) in different parts of the genome in *HDC1c* and *hdc1-1* (in % of all H3K9K14 regions). DARs were primarily located in transcription start site (TSS) regions and upstream gene. **(B)** Summary of the number of regions with more (hyper-acetylated, right) or less (hypo-acetylated, left) acetylated H3K9K14 comparing treatments (salt/control) or genotypes (*HDC1c/hdc1-1*) as indicated. Using ≥1.5 fold as cut-off, 7 DARs were identified in control conditions between *hdc1-1* and HDC1c. For seedlings grown on salt the number of *hdc1-1*/HDC1c was 117. In addition, the number of salt/control DARs was 521 in *hdc1-1* and 139 in HDC1c. **(C)** Venn diagrams depicting the number of common and unique differentially expressed targets (salt/control) between RNAseq and ChIPseq datasets for *HDC1c* and *hdc1-1*.

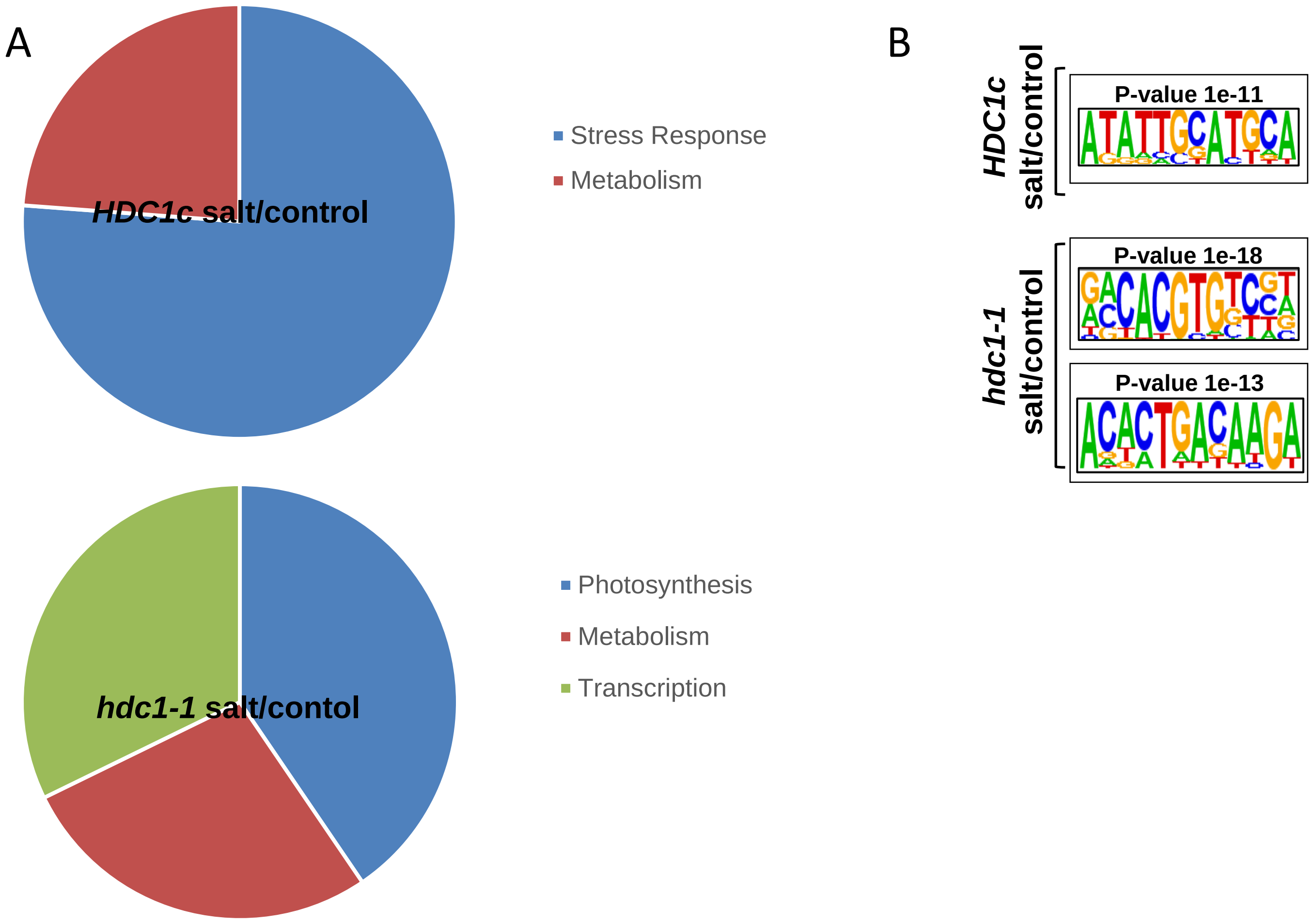

Fig. S9. **(A)** Gene ontology analysis of the ChIP targets identified in HDC1c and *hdc1-1* salt over control conditions (salt/control). Gene ontology analysis of the salt/control DARs in HDC1c showed enrichment for ABA-responsive proteins, calcium binding proteins, members of LEA family, annexins, ABRE-elements containing genes and cell-wall modifying enzymes such as xyloglucan endotransglucosylase/hydrolases (XTHs). The salt/control DARs in *hdc1-1* were enriched for transcription factors including DREB2A, AIL7, DBPF4, C2H2 and CCCH zinc finger protein members, as well as HD-ZIP leucine zipper proteins and NAC DOMAIN CONTAINING PROTEINs. **(B)** Enrichment for *de novo* motifs identified by Homer within promoter and TSS regions of differentially acetylated targets in HDC1c and *hdc1-1* salt over control (salt/control) comparisons. Analysis of consensus motifs enriched within promoter-localised salt/control DARs in HDC1c identified over-representation of the Ry-motif CATGCA(TG) that is present in many seed specific promoters (Mönke et al., 2004). For *hdc1-1* it was revealed an over-representation of the canonical G-box (CACGTG), a variation of an E-box and W-box (TGACA) motif which serves as a binding site for the members of the stress-induced WRKY transcription factor family (Ezer et al., 2017; Dhatterwal et al., 2019).

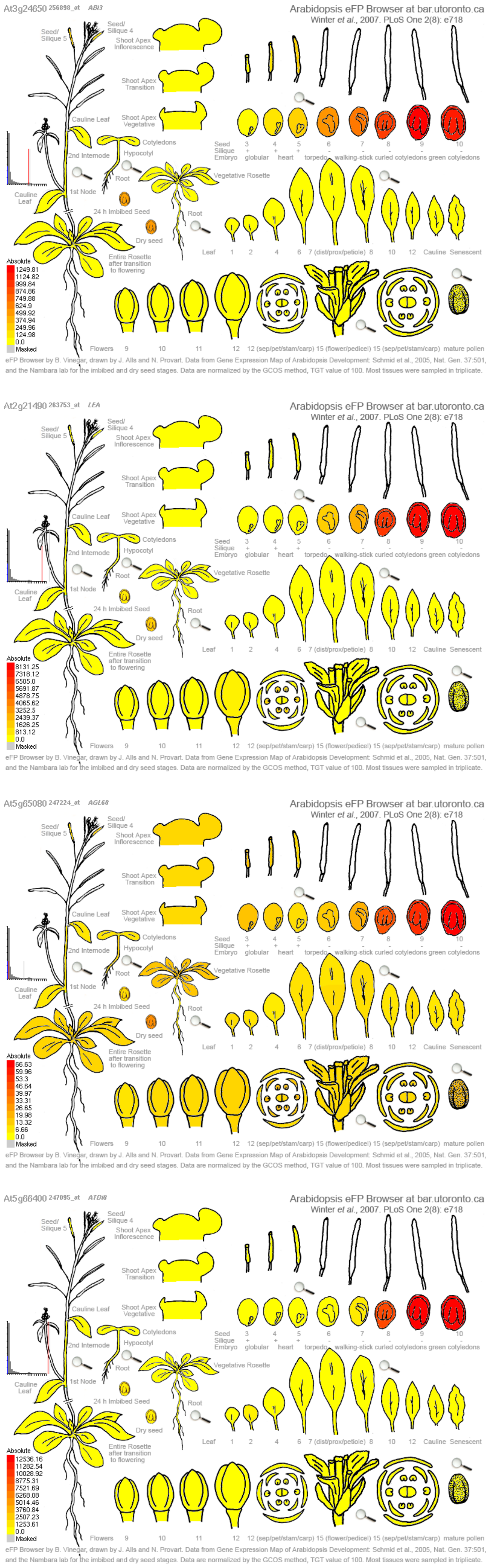

Fig. S10 Developmental expression levels of *ABI3, LEA, MAF5* and *RAB18* as recorded by the EFP browser (Winter et al., 2007; https://bar.utoronto.ca/efp/cgi-bin/efpWeb.cgi)

A

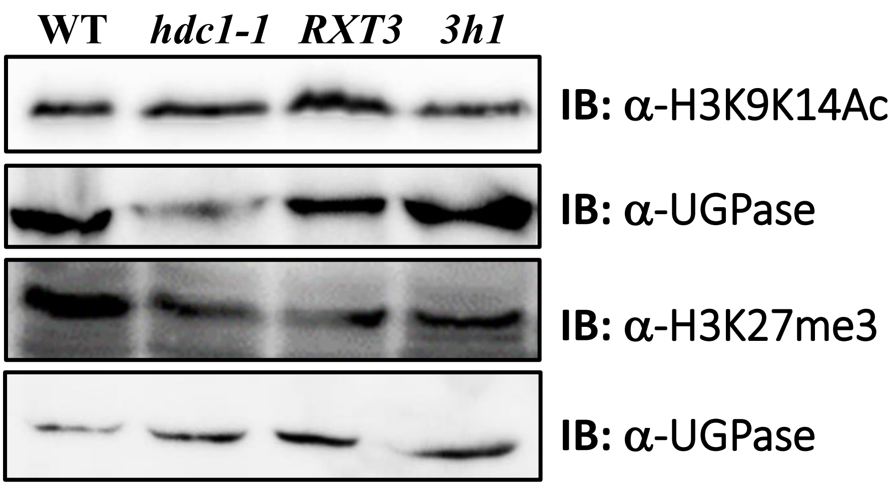

B

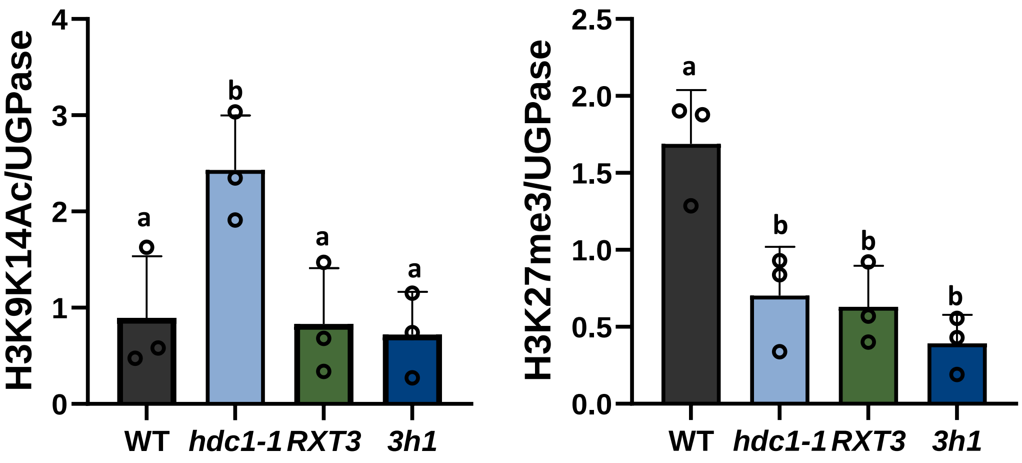

Fig. S11 (A) Detection of acetylated (a-H3K9K14Ac) (top) and methylated (H3K27me3) (bottom) histone 3 (a-H3) in protein extracts of wild type, *hdc1-1* mutant, *hdc1-1* overexpressing RXT3 domain *(RXT3)* and *3h1* mutant. For histone extraction three days old seedlings were grown on control plates (1/2 MS medium and 1% sucrose) supplemented with 100 mM NaCl. For all the experiments ~150 seedlings were grown in constant white light (75 mmol m^{-2} s^{-1}). Anti-UGPase was used as loading control. **(B)** Quantification of acetylated and methylated histone levels in wild type, *hdc1-1* mutant, *hdc1-1* overexpressing RXT3 domain *(RXT3)* and *3h1* mutant seedlings grown as above-reported. Bars are means of 3 experiments ± SD (reported above the bars). Letters indicate differences at $p < 0.05$ (one-way ANOVA).

A

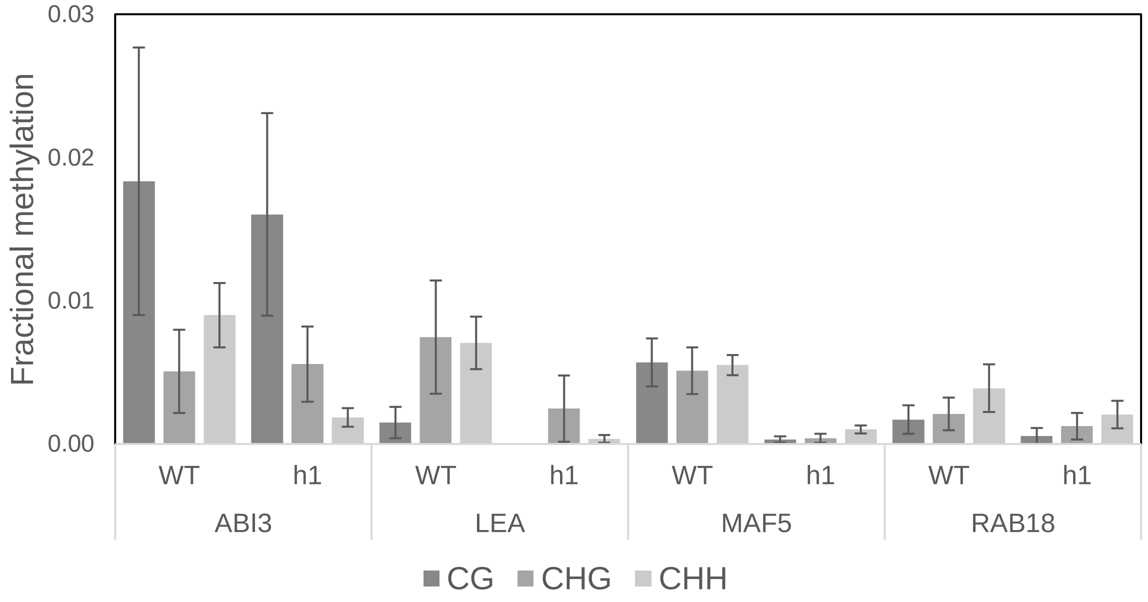

B

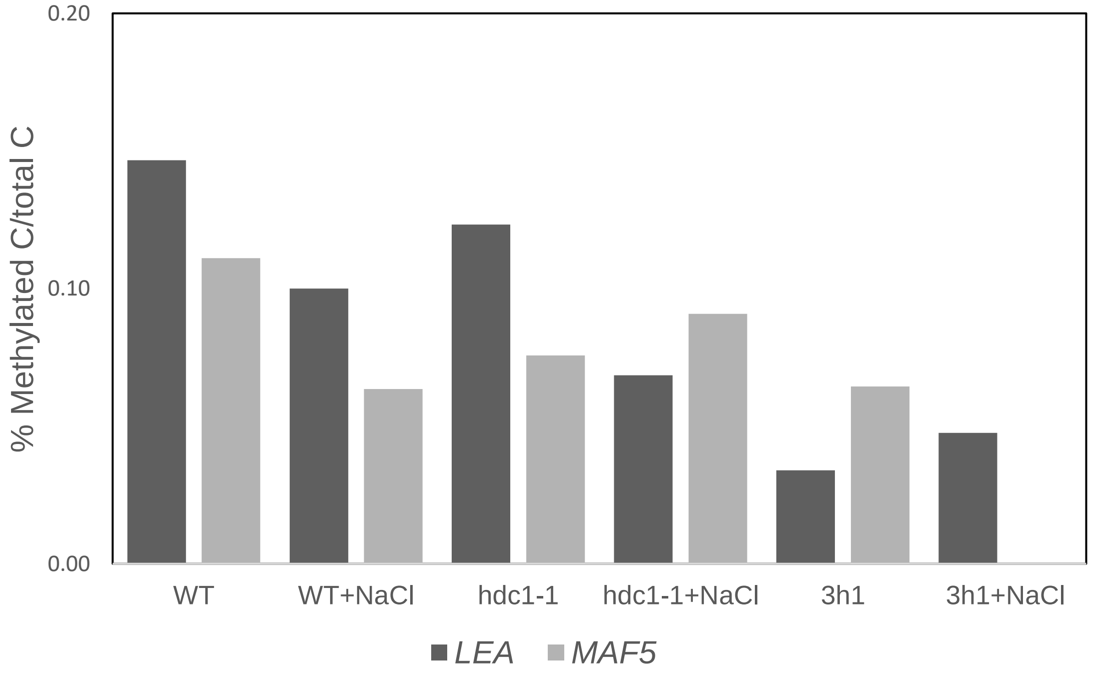

Fig. S12 (A) DNA methylation distribution across *ABI3*, *LEA*, *MAF5* and *RAB18* loci expressed in fractional methylation [#C/(#C+#T)] in wild type and *h1* mutant based on Bisulfite sequencing published in Zemach et al., 2013 (Note: the *h1* line in this publication is a *h1.1/h1.2* double mutant) . Bars are means **±** SD. **(B)** Percentage of methylated cytosines (C) of *LEA* and *MAF5* DNA regions spanning promoters and TSS in wild type, *hdc1-1* and *3h1* mutants of seedlings grown on control plates (1/2 MS medium) and control plates supplemented with 100 mM NaCl. For all the experiments ~150 seedlings were grown in constant white light (75 mmol m^{-2} s^{-1}) and samples were harvested at day 3. A minimum of five to eight clones per genotype and per treatment were sequenced.

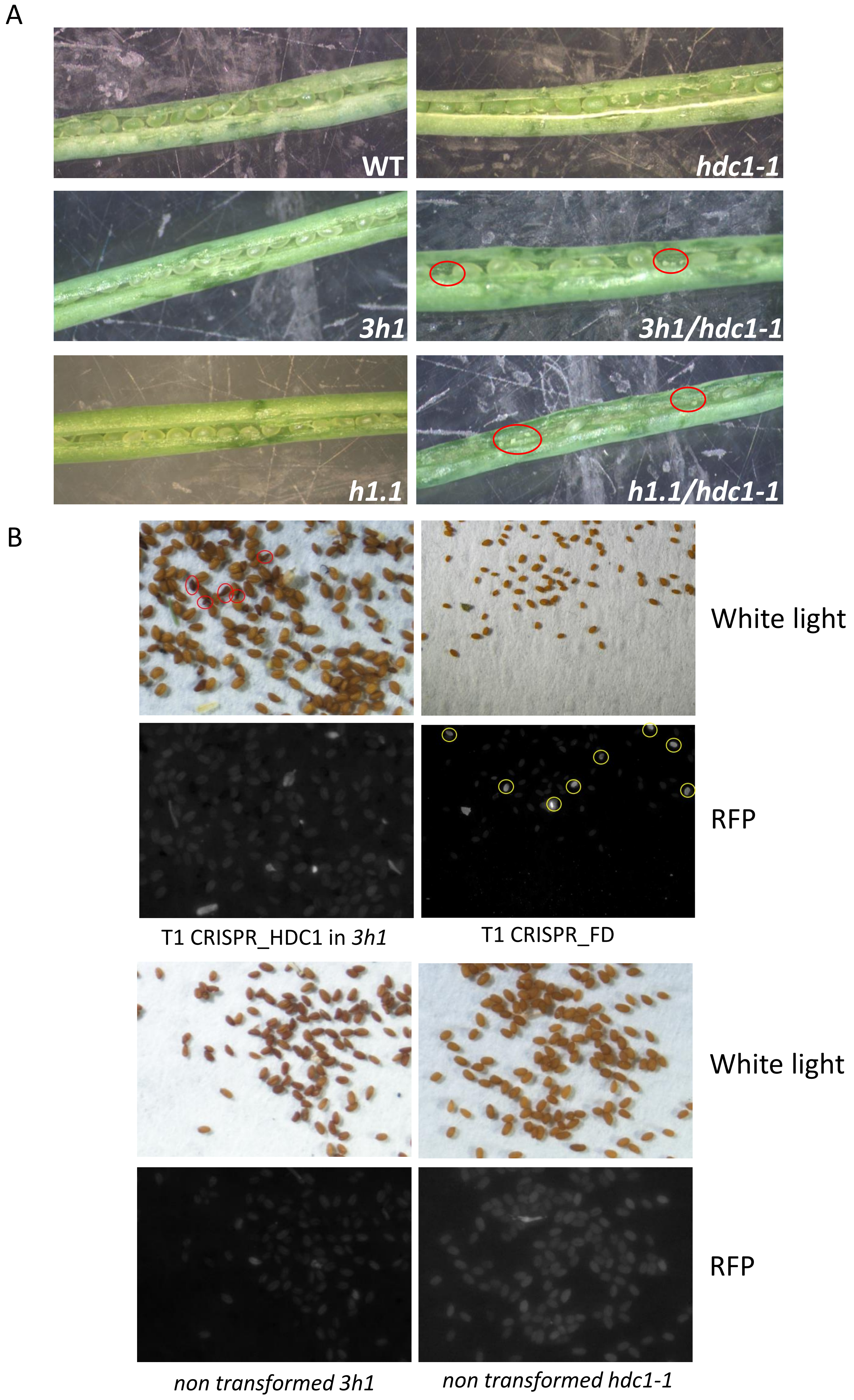
A
WT
hdc1-1
3h1
3h1/hdc1-1
h1.1
h1.1/hdc1-1
B
White light
RFP
T1 CRISPR_HDC1 in 3h1
T1 CRISPR_FD
White light
RFP
non transformed 3h1
non transformed hdc1-1

Fig. S13: (A) Siliques of wild type, *hdc1-1*, triple H1 (*3h1*) mutant, *h1.1* single mutant and putative quadruple and double mutants by crossing with *hdc1-1*. Red circles highlight aborted seeds within the siliques. **(B)** CRISPR Cas9 experiment to silence HDC1 in triple h1 (*3h1*) mutant. *3h1* plants were flower-dipped with a construct containing gRNAs that would knock-out HDC1. The construct also contains an RFP to allow screening for transformation based on fluorescence signal. Top left : Screening of T1 CRISPR_HDC1 in *3h1* shows no transformed seeds in the pods (lack of RFP signal) for >1000 tested seeds. Instead, a significant number of aborted seeds are observed (36 aborted seeds, 22.3% of the total in the picture) indicating that any successful transformation was lethal. Examples of aborted seeds are shown in the red circle. Top right: Independent transformation with CRISPR_FD showing the efficiency of transformation (see yellow-circled seeds). Bottom: Seeds of *3h1* and *hdc1-1* mutants showing no obvious aborted seeds.

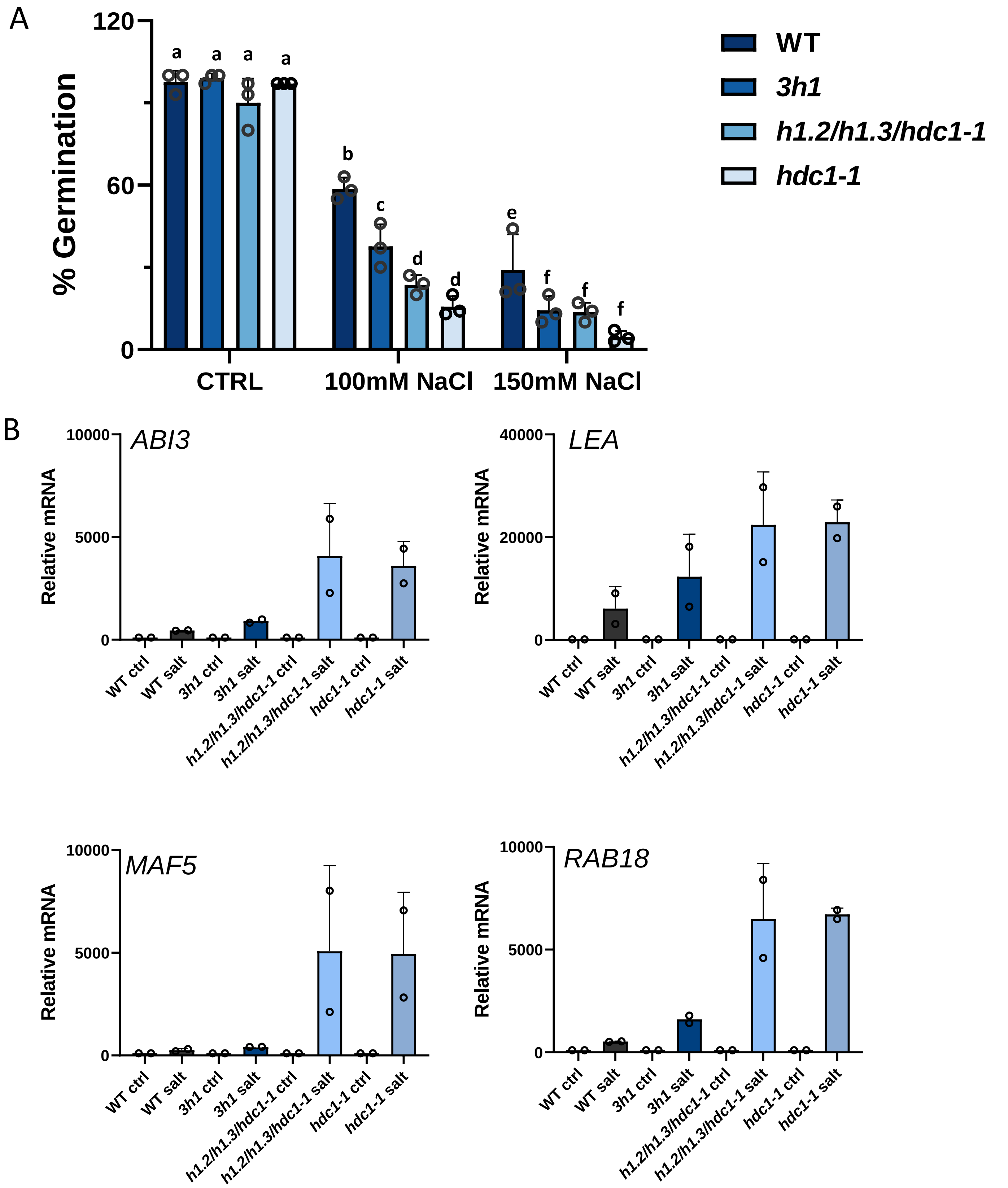

Fig. S14 (A): Germination rate of wild type (WT), knockout mutants of HDC1 and (*hdc-1-1*), triple knockout mutant of histone-1 variants (*3h1*) and triple knock mutant of histone H1.2, H1.3 variants and HDC1 (*h1.2/h1.3/hdc1-1*) Letters indicate differences at $p < 0.05$ (one-way ANOVA). (**B) :** mRNA levels of *ABI3*, *LEA*, *MAF5* and *RAB18* in 3-days old seedlings of wild type, *hdc1-1, 3h1* and *h1.2/h1.3/hdc1-1* seedlings grown on control or salt media determined by qPCR and normalised to the housekeeping gene *ISU1* (AT4G22220). Bars are means of 2 experimental replicates ± SD (reported above the bars).

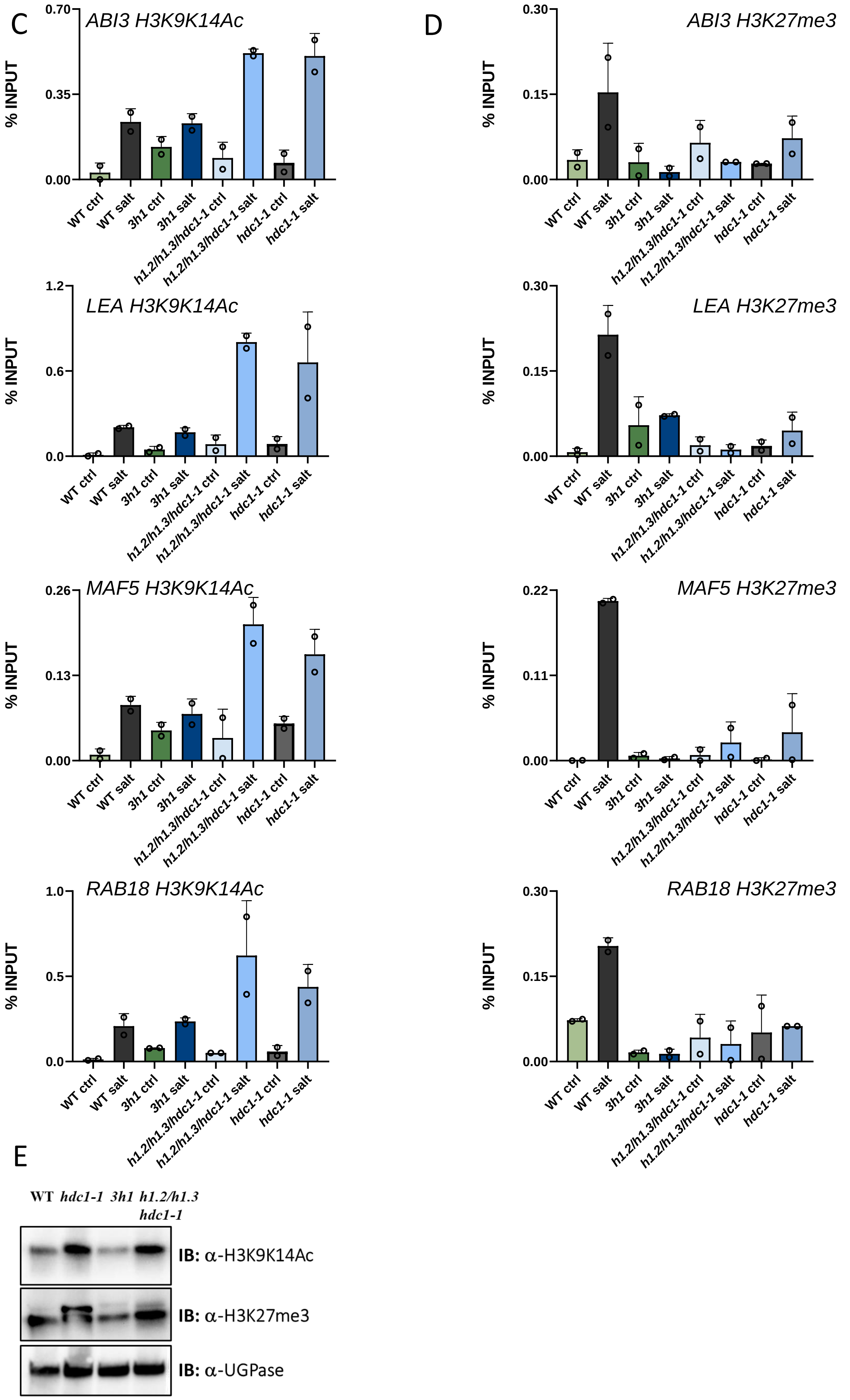

(C): H3K9K14Ac levels (in % of Input) of *ABI3, LEA*, *MAF5* and *RAB18* in wildtype (WT), *3h1, h1.2/h1.3/hdc1-1* and *hdc1-1* 3 days-old seedlings grown on control or salt media, determined by anti-H3K9K14Ac-ChIP qPCR. Bars are means of 2 experimental replicates ± SD (reported above the bars). (**D):** H3K27me3 levels (in % of Input) of *ABI3, LEA*, *MAF5* and *RAB18* 3-days old seedlings of wildtype (WT), *3h1, h1.2/h1.3/hdc1-1* and *hdc1-1* grown on control or salt media, determined by anti-H3K27me3-ChIP qPCR. Bars are means of 2 experimental replicates ± SD (reported above the bars). **(E):** Detection of acetylated (a-H3K9K14Ac) and methylated (H3K27me3) histone 3 (a-H3) in protein extracts of WT, *hdc1-1, 3h1, h1.2/h1.3/hdc1-1.* Immunoblot with anti-UGPase was used as loading control.

Primers for genotyping gDNA

Amplicon	Forward Primer	Reverse Primer
hdc1-1	CAAGGACTGGTGCTGAGAAAG	GCAGCCAAAATCTCAAGTAGC
maf5	CGTTTACGTCTCTTGGAAACG	TGGGTTTCACCAGAAAGTGTG
lea	AAAAATGATCTGGCTGTGCAC	ACGGTTTTATAGAAAAGGCGC
Lbb1-3	ATTTTGCCGATTTCGGAAC	pairs with mutant flanking sequence reverse primer
PAC161	TGATCCATGTAGATTTCCCG	pairs with GABI-KAT mutant flanking sequence reverse primer
h1.1	TTGAAATCCCACGTTTATTGG	GGGAGTTTAAACGAGGCTTTG
h1.2	TCTTTGGTCGGATTCAACAAC	TTCTTAGTTCCTTTAGCCGCC
h1.3	GAAAACCACCACTCATCCTCCATACTTTCA	CCTTCTTCACTTTCCTCTTCTTTTCTTGTTTGTTC
Ds5-4_R	TACGATAACGGTCGGTACGG	pairs with h1.3 primer

Primers for RT-qPCR

Amplicon	Forward Primer	Reverse Primer
ABI3	TTCCTCAGCTGCTTCTTGGG	GCACCAGAAGAGTCGTCACA
EIN3like	CCGTTATCTCTTCGCCGTCA	CCATTTTCTTGCGCCGTGAA
LEA	AGAAGTTCGGTAGCGGCAAA	GTTATGGTGGCCTGGAAGCT
MAF5	ACAGGGGATGAAAGAGCAGT	ACTTGAGAAGCGGGAGAGTC
ISU	GCCATCGCTTCTTCATCTGTTGC	TGGGAGAGAAAGATGCTTTG CG
RAB18	GGAGAAGTTGCCAGGTCATC	ACCGGGAAGCTTTTCCTTGATC
SAUR74	GGCCAGGAGAGATCCACAAG	ACGTCCAACCAACACTGTCT

Primers for ChIP qPCR

Amplicon	Forward Primer	Reverse Primer
ABI3	TTCCTCAGCTGCTTCTTGGG	GCACCAGAAGAGTCGTCACA
EIN3like	CCGTTATCTCTTCGCCGTCA	CCATTTTCTTGCGCCGTGAA
LEA	CCGGTGTTGTTAGCTCCACT	CCAACGGTGCTAGTAGGGTG
MAF5	AGGGATCGGCGTAGAGTTCT	TGTCTTCAAGATCCTGCCATGA
RAB18	CGTCTTACCAGAACCGTCCA	CCGTATCCTCCTCCTCCCAT
SAUR74	TGCTCATCTCTATCTCTCCCCT	TCACTAACCTGTCGCCTGTC
ACTIN	CGTTTCGCTTTCCTTAGTGTTAGCT	AGCGAACGGATCTAGAGACTCACCTTG
H3K9me2 1-1	AAGAGAGCTGGCAGAAGCAGTTGA	ACGCCCTTTACCTTGACCTCCTTT
H3k9me2 1-2	TGTGTGGAAGGGTCTTGTGGACTT	AACTTACATGTTTGCGGGCACGAG

Primers for CRISPR-Cas9

Amplicon	Forward Primer	Reverse Primer
pKItRNA_AarI	TCACCTGCCCCCATTGAACAAAGCACCAGTGGTCTA	TTCACCTGCCCCCAAACTGCACCAGCCGGGAATCG
gRNA HDC1 11	ATTGAGGGTGAAGGAGCTACCGAG	AAACCTCGGTAGCTCCTTCACCCT
gRNA HDC1 65	ATTGCGACTGCAAATGTGGACCCG	AAACCGGGTCCACATTTGCAGTCG

Primers for Bisulfite sequencing

Amplicon	Forward Primer	Reverse Primer
LEA	GGTTTTAAGTTTTGATTAAATTTATATGTG	TCCAATACCAACAAAAACTCCTATC
MAF5	GGTTTTGAGGGTTAGAATTTTAGAAAT	CATAATCCAAAATCAAACAAAATCC

Table S1: Primer sequences used in this study.
